# The histone demethylase Kdm3 prevents auto-immune piRNAs production in *Drosophila*

**DOI:** 10.1126/sciadv.ade3872

**Published:** 2023-04-07

**Authors:** Karine Casier, Julie Autaa, Nathalie Gueguen, Valérie Delmarre, Pauline P. Marie, Stéphane Ronsseray, Clément Carré, Emilie Brasset, Laure Teysset, Antoine Boivin

**Affiliations:** ^1^ Transgenerational Epigenetics and Small RNA Biology, Sorbonne Université, CNRS, Institut de Biologie Paris-Seine, Laboratoire Biologie du Développement, UMR7622, F-75005 Paris, France.; ^2^ iGReD, CNRS, INSERM, Faculté de Médecine, Université Clermont Auvergne, 63000 Clermont-Ferrand, France.

## Abstract

Genome integrity of the animal germline is protected from transposable element activity by PIWI-interacting RNAs (piRNAs). While piRNA biogenesis is intensively explored, little is known about the genetical determination of piRNA clusters, the genomic sources of piRNAs. Using a bimodal epigenetic state piRNA cluster (*BX2*), we identified the histone demethylase Kdm3 as being able to prevent a cryptic piRNA production. In the absence of Kdm3, dozens of coding gene-containing regions become genuine germline dual-strand piRNA clusters. Eggs laid by *Kdm3* mutant females show developmental defects phenocopying loss of function of genes embedded into the additional piRNA clusters, suggesting an inheritance of functional ovarian “auto-immune” piRNAs. Antagonizing piRNA cluster determination through chromatin modifications appears crucial to prevent auto-immune genic piRNAs production.

## Teaser

Incorrect determination of small RNA sources through chromatin remodeling in female germline leads to homeotic defects in embryo.

## INTRODUCTION

In animals, germ line genome integrity is preserved by small, noncoding, dedicated RNAs acting as an immune system against transposable element (TE) activity and called PIWI-interacting RNAs (piRNAs) (*1*, *2*). In *Drosophila*, piRNA production is initiated from heterochromatic loci containing remnants of TEs and enriched in histone H3 trimethylated on lysine 9 (H3K9me3) (*3*–*5*). These loci, called piRNA clusters, constitute a memory of past TE invasions. Two types of piRNA clusters coexist in the *Drosophila* gonads: those that are transcribed from a canonical RNA polymerase II promoter, producing single-strand RNA precursor and so-called uni-strand piRNA cluster, (*3*, *6*, *7*) and those whose transcription lacks defined promoters giving rise to transcripts from both DNA strands (*3*, *6*, *8*–*11*). These latter are called dual-strand piRNA clusters and are germline specific. How germinal dual-strand piRNA clusters are defined and activated at each generation remains elusive: Repetitive DNA sequences, specific chromatin marks, flanking transcription units, and maternal inheritance of homologous piRNAs are all features that appear critical for their determination (*11*–*14*). One of the key determinants of germinal piRNA clusters is Rhino, a homolog of the heterochromatin protein 1a (HP1a) (*8*, *15*). Rhino specifically binds the H3K9me3-enriched chromatin of piRNA clusters through its N-terminal chromodomain (*6*, *14*, *16*) and recruits proteins allowing a noncanonical transcription of piRNA clusters (*6*, *11*), the maturation, and the nuclear export of the piRNA precursors (*9*, *10*, *17*–*21*). How Rhino selectively recognizes H3K9me3-enriched piRNA clusters and not other H3K9me3-enriched regions, such as the pericentric heterochromatin, remains to date unexplored. Maternally inherited piRNAs are currently thought to be the sequence-specific signal that target Rhino to the transcripts coming from H3K9me3-enriched regions in the zygote, thus maintaining, throughout generations, the memory of piRNA cluster identity (*6*, *13*, *14*). However, how piRNA clusters are originally defined and start to produce piRNAs is still not understood. To further elucidate mechanisms that define germinal piRNA clusters, we performed an original genetic screen revealing genes involved in maintaining *BX2*, a repetitive locus that can produce piRNAs (ON state) (*13*, *22*, *23*), in an inactive piRNA production state (OFF state). Here, we reveal that the histone demethylase Kdm3 prevents *BX2* and a dozen of other gene-containing regions from becoming bona fide germinal dual strand piRNA clusters. Eggs laid by *Kdm3* mutant females show developmental defects that phenocopy loss of function of genes embedded into the additional piRNA clusters. This can suggest an inheritance of functional ovarian “auto-immune” piRNAs.

## RESULTS

### Germline knockdown of *half pint* or of *Kdm3* activates a piRNA cluster without homologous piRNA maternal inheritance

**Fig. 1.**
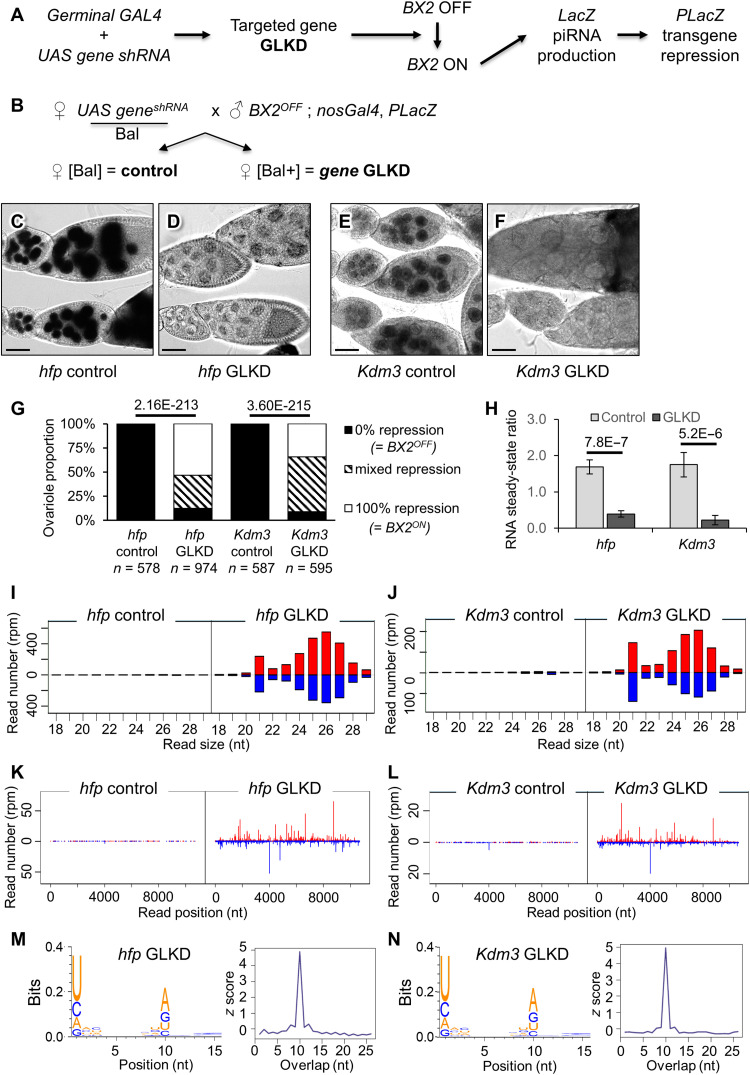
*hfp* and *Kdm3* GLKD lead to de novo activation of the *BX2* piRNA cluster. (**A**) Strategy followed to identify genes required to maintain *BX2* in an inactive state (*BX2*^OFF^). (**B**) Genetic scheme performed to compare the *BX2* ON/OFF state between GLKD and control contexts. Bal is for a balancer carrying a dominant phenotypic marker. (**C** to **F**) β-Galactosidase staining of ovaries. The corresponding genotype is indicated below each picture. Scale bars, 30 μm. (**G**) Ovariole repression was measured by counting the number of ovarioles showing no, partial, or complete repression of the *P(lacZ)* target among egg chambers. *P* values are indicated above bars (Pearson’s χ^2^ test). *n* is the number of ovarioles counted. (**H**) Measurement of *hfp* and *Kdm3* RNA steady-state level by RT-qPCR from control and respective GLKD ovaries. *n* = 5 biological replicates for each condition. Error bars are SD. *P* values are indicated above bars (bilateral *t* test). (**I** and **J**) Comparison of the size distribution of 18- to 29-nt ovarian small RNAs (sRNAs) matching *BX2* sequences between *BX2* control (*BX2*^OFF^) and *BX2* activated by *hfp* GLKD (I) or by *Kdm3* GLKD (J). Positive and negative values correspond to sense (red) and antisense (blue) reads, respectively. (**K** and **L**) Comparison of 23- to 29-nt unique mappers along the *BX2* sequence between *BX2* control (*BX2*^OFF^) and *BX2* activated by *hfp* GLKD (K) or *Kdm3* GLKD (L). (**M** and **N**) Logo showing the enrichment in a U at the first position, 66.8% 1U, *n* = 28,029 reads in *hfp* GLKD ovaries (M) and 72.0% 1U, *n* = 11,806 reads in *Kdm3* GLKD ovaries (N) and the enrichment of an A at the 10th position among the paired reads (49.3% 10A, *n* = 2237 for *hfp* GLKD (M) and 57.7% 10A, *n* = 929 for *Kdm3* GLKD (N). The 5′ overlap probability *z* score of paired reads is shown for *hfp* and *Kdm3* GLKD, respectively.

Using *BX2*, a locus made of seven *P-lacZ-white* [*P(lacW)*] transgenes and behaving as a dual-strand piRNA cluster (*13*), we previously showed that an environmental stress can activate de novo *BX2*, likely through an increase of its antisense transcription (*23*). Using the bimodal epigenetic state ability of *BX2*, which can be in an ON or an OFF state for piRNA production, we designed a genetic screen allowing us to test the effect of germline knockdown (GLKD) of genes on *BX2* activation (Fig. 1, A and B). We tested 491 small hairpin RNA (shRNA)–producing lines (*24*) affecting 341 candidate genes mainly involved in RNA biology and chromatin remodeling (table S1). Among those, only two independent lines enabled the conversion of *BX2*^OFF^ into *BX2*^ON^, as shown by its ability to functionally repress in trans a homologous euchromatic *P(lacZ)* target (Fig. 1, C to G). The first line is designed to specifically target the splicing factor *half pint* (*hfp*) and the second line targets specifically *Kdm3*. *Kdm3* encodes a Jumonji C (JmjC) domain–containing histone demethylase, the single *Drosophila* ortholog of mammalian KDM3A and KDM3B that catalyze H3K9me2 demethylation (*25*). KDM3B is involved in gene activation in leukemia cells (*26*, *27*); for review, see (*28*). In *Drosophila*, Kdm3 was found in substantially increased association with mutated H3K9M-containing mono-nucleosomes (*29*). In addition, its loss of function has been reported to act as an enhancer of position effect variegation (PEV) (*30*), while its overexpression acts as a suppressor of PEV (*29*), suggesting that Kdm3 acts as a structural antagonist to heterochromatin. PEV is a phenomenon that occurs when a euchromatic sequence is relocated next to heterochromatic regions (*31*). The expression of relocated genes is then subject to stochastic and bimodal (ON/OFF) expression due to the propagation of heterochromatin proteins, such as HP1a, overflanking sequences to varying degrees from cell to cell. This cell-autonomous phenomenon thus produces a variegated phenotype. We previously observed such an incomplete, bimodal, and stochastic repression of the *P(lacZ)* target in the adult ovaries resembling to variegation (*23*, *32*), referred to as “mixed repression” in this study. We showed previously that it can occur at all developmental stages (*33*). The interesting similitude between incomplete piRNA-mediated repression and PEV phenotypes suggested the role of chromatin remodeling factors in both mechanisms long before their functional discovery.

GLKD efficiencies were checked by reverse transcriptase followed by quantitative polymerase chain reaction (RT-qPCR) on total ovarian RNA (Fig. 1H) and their effect on *BX2* conversion were confirmed with characterized *hfp* and *Kdm3* mutant alleles. While amorphic or strong alleles of *hfp* lead to homozygous embryonic lethality, *hfp*^9^ and *hfp*^13^ are two hypomorphic alleles that allow adult survival (*33*). Different allelic mutant combinations led to the conversion of *BX2*^OFF^ into *BX2*^ON^, confirming the specific role of *hfp* in this process (fig. S1, A to F). Similarly, a trans-heterozygous combination of null alleles of *Kdm3* (*30*, *34*) led to the *BX2* conversion confirming the role of *Kdm3* in the *BX2*^OFF^ state maintenance (fig. S1, G to I). Whole-sequencing analyzes of small RNAs (sRNAs) extracted from controls, *hfp*, and *Kdm3* GLKD ovaries revealed that, in agreement with the functional silencing of the *P(lacZ)* target, a characteristic profile of *BX2* locus–derived sRNAs composed of a peak of 21–nucleotide (nt)–long [corresponding to small interfering (siRNAs)] and a bulk of 23- to 29-nt-long (corresponding to piRNAs) was produced in both *hfp* and *Kdm3* GLKD ovaries when compared to their respective control sisters (Fig. 1, I and J and table S2). It was recently reported that siRNAs can provide initial trigger to activate some piRNA clusters (*35*). To test the relevance of siRNAs in the activation of *BX2* in an *hfp* or *Kdm3* GLKD, we reproduced the above experiment in a *Dicer-2* (*Dcr-2*) homozygous loss-of-function mutant context. Dcr-2 is a ribonuclease (RNAse) III enzyme that is required for siRNA production (*36*) and we have previously shown that in a *Dcr-2^L811fsX^* homozygous context, 21-nt-long RNA production from *BX2* locus was abolished (*22*). We show here that the activation of *BX2* by *hfp* GLKD or *Kdm3* GLKD was not altered in *Dcr-2^L811fsX^* homozygous mutant (fig. S2, A and B) demonstrating that, in this case, siRNAs are not required for *BX2* activation. We therefore focused on 23- to 29-nt sRNAs that map all along the *BX2* sequence (Fig. 1, K and L). These sRNAs were enriched with a U at 5′ end and present, among the paired reads, an enrichment of an A at the 10th position known as the ping-pong signature confirmed by calculation of the 5′ overlap probability *Z* scores (Fig. 1, M and N) (*3*). Together, our results show that *hfp* or *Kdm3* GLKD induces piRNA production and trans-silencing capacities of the *BX2* locus, demonstrating that a locus made of repeated sequences could require a genetically active process to maintain a non–piRNA-producing state (OFF).

### The *BX2* conversion is stable through generations

*BX2 hfp* GLKD flies are fertile, allowing us to check the stability of the *BX2* OFF-to-ON conversion in following generations and after a return to a wild-type dosage of *hfp* (fig. S3A). Ovarian sRNA analyses revealed that the progeny of *BX2 hfp* GLKD flies that do not produce shRNA against *hfp* anymore however maintained the production of numerous piRNAs matching *BX2* sequence when compared to the progeny of *BX2* control flies (fig. S3, B to D). These piRNAs are enriched in U at the 5′ end and in A at the 10th position among the paired reads (fig. S3E). The *BX2*^ON^ state was fully maintained in seven independent lines established from seven single G2 *BX2* females and tested during 20 generations (table S3). These results demonstrate that, once established by a transient reduction of the *hfp* dosage, the acquired *BX2*^ON^ state is maintained through generations likely by maternal inheritance of *BX2* piRNAs (*13*, *22*). In contrast to *hfp* GLKD flies, *Kdm3* GLKD and *Kdm3* KO flies do not produce viable progeny, preventing us to analyze the stability of this conversion in subsequent generations.

### Mutants of *otu* do not activate *BX2*

To further investigate the mechanism of *BX2* conversion by *hfp* GLKD, we first checked whether the direct down-regulation of a known target of Hfp could activate *BX2*^OFF^. The *ovarian tumor* gene (*otu*) encodes a deubiquitinase involved in several processes including germ cell development. *Otu* sounded to be a good candidate because the specific 104-kDa isoform, which is produced through the Hfp-mediated splicing (*33*), encodes a TUDOR domain that is shared by a lot of proteins involved in piRNA biology (Spn-E, Krimp, Qin, Tej, Tapas, Vret, Tudor, SoYb, BoYb, and Yb) and conserved in most animals (*2*). Therefore, we tested the effect of *otu* GLKD or *otu* mutant alleles on *BX2*^OFF^ conversion. The *otu* GLKD context leads to atrophic gonads with few egg chambers and no *BX2* activation (*n* = 15 females). Coherently, two allelic mutant combinations of *otu* did not induce *BX2* conversion either (fig. S3F). Together, these results demonstrate that the *hfp* GLKD effect on *BX2* conversion does not rely on *otu* splicing regulation nor on the 104-kDa isoform production.

### Hfp regulates *Kdm3* splicing

**Fig. 2.**
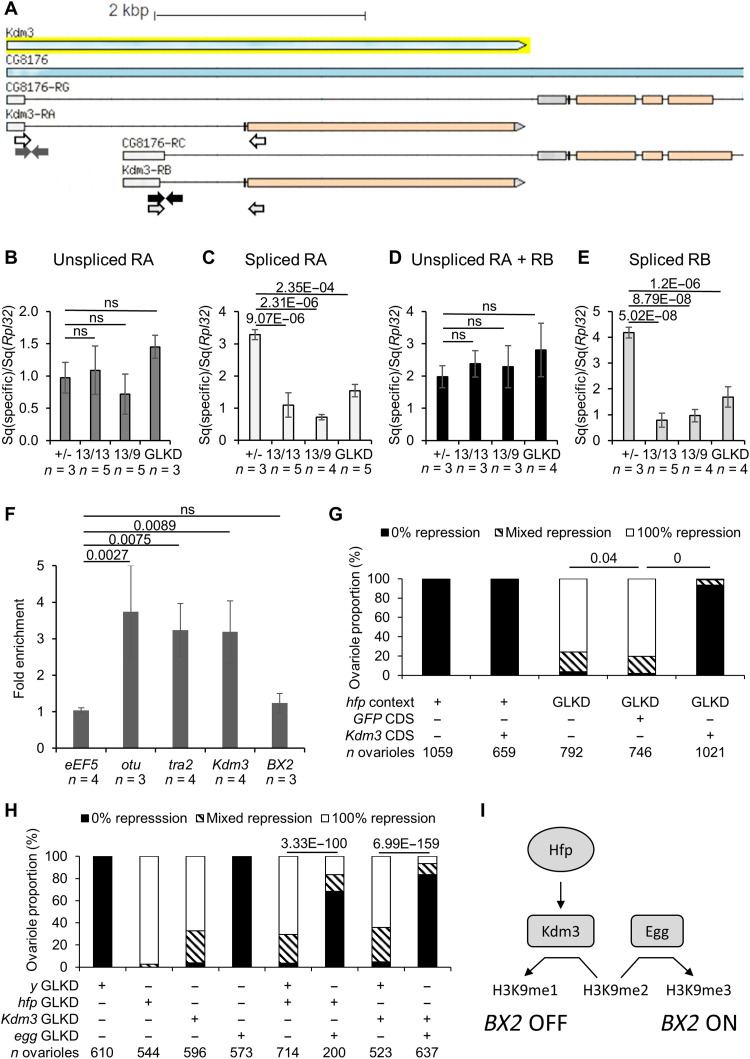
Interactions between *hfp*, *Kdm3* and *egg* in *BX2* conversion. (**A**) The two *Kdm3* RNA isoforms (*RA* and *RB*) share a 5′UTR (light gray boxes) with two RNA isoforms of *CG8176*. Colored arrows indicate primers used for following RT-qPCR experiments. (**B** to **E**) RT-qPCR experiments using specific primers shown in 3a and *Rpl32* primers as reference. Sq, standard quantity. *n* is the number of biological replicates. Error bars are SD. *P* values are indicated above bars [analysis of variance (ANOVA) following by Tukey post hoc test, ns (nonsignificant) is for *P* value >0.05]. (**F**) RNA immunoprecipitation (RIP) experiment using a *hfp-GFP* construct. Error bars are SD. *P* values are indicated above bars (ANOVA following by Tukey post hoc test, ns is for *P* value >0.05). (**G**) Rescue experiment using Kdm3 CDS. Histogram shows the percentage of ovarioles showing homogeneous β-galactosidase staining in all egg chambers (0% repression, *BX2*^OFF^**), homogeneous nonstaining in all egg chambers (100% repression, *BX2*^ON^**) or mixed repression. For all crosses, males bore *BX2*^OFF^**, *nosgal4*, and *P(lacZ)* transgenes and females carried a combination of *UAS-hfp shRNA* (*hfp* GLKD), *UASpRFP* (as control of a potential GAL4 dilution effect on two UAS transgenes), and/or *UASpKdm3* CDS transgenes. *P* values are indicated above bars (Pearson’s χ^2^ test). *n* is the number of counted ovarioles. (**H**) Epistatic relationships between *hfp*, *Kdm3*, and *egg* GLKD upon the activation of a paternally inherited *BX2*. Histogram shows the percentage of ovarioles as in (G). For all crosses, males bore *BX2*^OFF^**, *nosgal4*, and *P(lacZ)* transgenes, while females carried a combination of *UAS-hfp shRNA*, *UAS-Kdm3 shRNA*, *UAS-egg shRNA*, and/or *UAS-y shRNA* as a control for a potential GAL4 dilution effect on two UAS transgenes. *n* is the number of counted ovarioles. (**I**) Model of regulation of the epigenetic state of *BX2* by modulation of the methylated state of the lysine-9 of histone H3 (H3K9) by Kdm3 and Egg.

Given the annotated *Drosophila* genome, *Kdm3* shares its 5′ untranslated region (UTR) with the *CG8176* gene that encodes an F-BAR domain–containing protein potentially involved in cytokinesis [Fig. 2A and (*37*)]. This peculiar genomic position suggests that *Kdm3* expression relies on specific splicing factors. To test whether the production of *Kdm3* transcripts depends on Hfp, we measured by RT-qPCR the steady-state level of the two *Kdm3* isoforms RNA in different *hfp* mutant or *hfp* GLKD backgrounds. Four different primer sets revealed that both *Kdm3* RA and RB spliced forms are strongly affected in all tested *hfp* mutant and GLKD backgrounds, while unspliced isoforms were almost unaffected (Fig. 2, B to E). To investigate whether Hfp plays a direct role on *Kdm3* splicing, RNA immunoprecipitation (RIP) experiments were carried out using Hfp–green fluorescent protein (GFP)–expressing flies and anti-GFP antibodies followed by RT-qPCR analyses. As expected, RNAs from known target of Hfp such as *tra2* (*38*) and *otu* (*33*) were significantly enriched when compared to control gene (*eEF5*) (Fig. 2F). *Kdm3* RNA was also significantly enriched, supporting the fact that Hfp is directly involved in *Kdm3* splicing (Fig. 2F). We did not detect any interaction between Hfp and *BX2* transcripts arguing against a direct effect of Hfp on *BX2* conversion (Fig. 2F).

### *Kdm3* and *egg* act antagonistically on the *BX2* epigenetic state

To test whether the *BX2* conversion in *hfp* GLKD background functionally depends on *Kdm3*, we constructed a transgene containing the coding sequence (CDS) of *Kdm3* whose expression relies on the GAL4/UAS system (see Material and Methods). Using a driver expressing GAL4 in the germline, we showed that the germinal expression of *Kdm3* CDS prevents the *BX2* conversion in an *hfp* GLKD background (Fig. 2G). In that context, 93% of ovarioles (*n* = 1021) showed β-galactosidase expression in all egg chambers, thus demonstrating that *BX2* was not converted into an active piRNA cluster. This result strongly suggests that *Kdm3* splicing performed by Hfp is critical for *BX2* conversion. We then hypothesized that *BX2* is actively maintained OFF by Kdm3, an H3K9me2 demethylase enzyme that might counteract the function of an antagonist protein involved in the piRNA pathway to avoid piRNA cluster conversion. One of the best candidates is Eggless/SetDB1 (Egg), a H3K9 methyltransferase that has been shown to be required for germinal piRNA cluster determination (*5*, *39*, *40*). We have tested this hypothesis by analyses of epistasis relationship between *egg*, *hfp*, and *Kdm3* GLKD. The concomitant loss of *egg* and *hfp* on one hand or of *egg* and *Kdm3* on the other hand prevents *BX2* conversion (Fig. 2H). These results show that *egg* is required for the *BX2* epigenetic conversion in an *hfp* or *Kdm3* GLKD background very likely through H3K9 methylation level (Fig. 2I).

### Other transgenic clusters can be activated upon *hfp* or *Kdm3* GLKD

Previously, we have shown that the stability throughout generations of *P(lacW)* clusters activated by maternally inherited homologous piRNAs (paramutation) depends on the size of the cluster, i.e., the number of transgenes making the cluster (*13*). Here, we tested whether the number of *P(lacW)* transgenes present in the same locus influences the conversion efficiency in *hfp* or *Kdm3* GLKD backgrounds (fig. S4, A and B). As observed for the paramutation phenomenon, the longer the cluster, the higher the conversion rate (fig. S4C). Furthermore, smaller clusters (one and two copies) could not be activated for piRNA production, revealing that the conversion requires a minimum of four repeats and do not depend on the genomic integration location. Next, we asked whether a subtelomeric piRNA cluster could be stimulated for piRNA production in *hfp* or *Kdm3* GLKD backgrounds. Previously, we have described that the activation for piRNA production of transgenes embedded in subtelomeric piRNA clusters was spontaneous but delayed when the transgenes were paternally inherited (*32*). Four generations were required to reach a full production of piRNAs and a full repression of a *P(lacZ)* target in a β-galactosidase functional assay (*32*). Here, we show that *hfp* and *Kdm3* GLKD markedly increased subtelomeric transgene activation during the first generation (fig. S4, D and E). These results show that the activation effect is not restricted to the *BX2* piRNA cluster family. As the subtelomeric transgenes are embedded into natural piRNA clusters, it also suggests that Kdm3 may counteract the propagation of piRNA production capacity from neighboring piRNA clusters, thereby helping in defining the frontiers of piRNA clusters.

### Dozens of genomic regions become piRNA clusters upon *Kdm3* GLKD

To know whether other genomic regions could be actively turned ON for piRNA production, we performed a comparative analysis of sRNA production of three replicates from control and *Kdm3* GLKD ovaries. Despite its strong effect on the conversion of *BX2* family and subtelomeric piRNA clusters, *Kdm3* GLKD did not induce major changes in the global piRNA production in ovaries. The number of 23- to 29-nt RNAs mapping the *Drosophila* genome or previously defined piRNA clusters was not significantly modified (Table 1) (*3*, *41*). We noted a small but nonsignificant increase of piRNAs matching transposon sequences (Table 1), suggesting that a small number of 23- to 29-nt RNA genomic sources could have changed.

**Table 1.** Number of 23- to 29nt sRNA matching *Drosophila* genome, previously defined piRNA clusters and transposon sequences. Total or unique mappers are indicated. *n* is the mean number of reads given in read per million (rpm). SD is the standard deviation observed between the three biological replicates. *P* value from Mann-Whitney is given.

	Control		*Kdm3* GLKD		*P* value (Mann-Whitney)
	*n* (rpm)	SD	*n* (rpm)	SD	
*Drosophila* genome (total)	256,539	3987	269,805	8524	0.2
*Drosophila* genome (unique)	60,577	890	64,494	2783	0.1
piRNA clusters defined in (*3*) (unique)	55,841	939	56,665	1820	0.7
piRNA clusters defined in (*41*) (unique)	67,432	1078	67,807	839	0.7
Transposons (total)	174,550	5708	189,898	6001	0.1
Transposons (unique)	29,448	993	33,383	1078	0.1

**Fig. 3.**
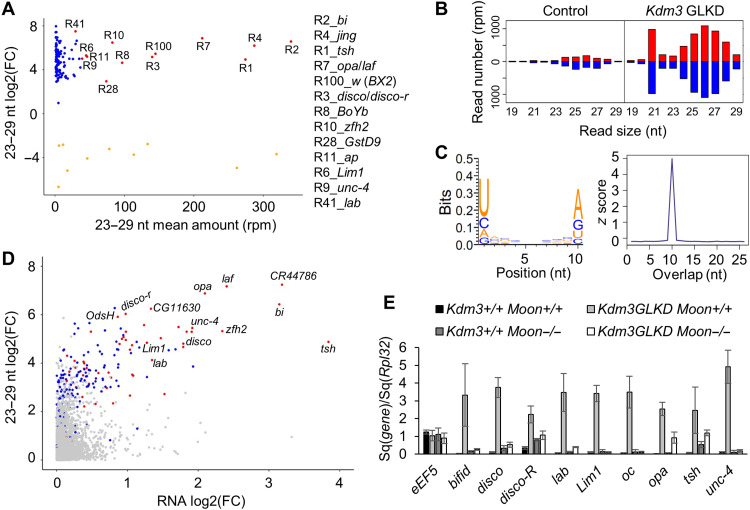
Characterization of additional piRNA clusters upon *Kdm3* GLKD. (**A**) Ovarian small RNAs (sRNAs) (23 to 29 nt, unique mappers per 1-kb bins) fold change (log2, *y* axis) between *Kdm3* GLKD and control compared to the mean amount of 23 to 29 nt (*x* axis) revealed that 123 gene-containing regions produce a significant increased amount of sRNAs (blue and red dots). Thirteen major regions (red dots) are listed with included major gene names. (**B**) Comparison of the size distribution of sRNAs (18 to 29 nt) matching the 13 major producing regions between control and *Kdm3* GLKD contexts. Positive and negative values correspond to sense (red) and antisense (blue) reads, respectively. (**C**) Logo showing the enrichment in U1 and A10 of the 10-nt paired sRNAs that match the 13 major producing regions in *Kdm3* GLKD and their 5′ overlap probability *z* score. (**D**) Ovarian sRNAs (23 to 29 nt, unique mappers on genes) fold change (log2, *y* axis) between *Kdm3* GLKD and control compared to RNA fold change between *Kdm3* GLKD and control (log2, *x* axis) from total RNA-seq. Red dots correspond to genes contained in the 13 most producing piRNA regions in *Kdm3* GLKD ovaries [same as (A)], dark blue dots are genes contained in the 123 additional producing regions, light blue dots are genes presenting a significant difference (*P* value <0.05), and gray dots are nonsignificant differentially expressed genes (*P* value >0.05). (**E**) RT-qPCR measurements using specific primers corresponding to tested genes and *Rpl32* primers as reference. They confirm the increase of RNAs observed by RNA-seq [in (B)] in *Kdm3* GLKD (light gray columns) compared to control (dark columns). This increase depends on Moonshiner (white columns). The loss of Moonshiner itself does not alter the RNA steady state level of these regions (dark gray columns). A complete statistical analysis is given on table S6.

To question the source of piRNA production, the *Drosophila* genome was first split into one kilobase (kb) bins, and the unique 23- to 29-nt reads isolated from control and *Kdm3* GLKD ovaries were then mapped on these bins. Bins producing differential amount of 23- to 29-nt RNAs (DESeq2 analysis, fold change ≥8, *P* value <0,001) were recovered. Selected bins distant from less than 3 kb were grouped resulting in 134 differentially 23- to 29-nt-expressing regions (table S4). They range from 6 to 182 kb and represent a total of 2.5 megabase (Mb). Eleven intergenic regions (totalizing 144 kb) produce less 23- to 29-nt RNAs in *Kdm3* GLKD than in control, while 123 regions (totalizing 2.38 Mb) produce significantly more 23- to 29-nt RNA in *Kdm3* GLKD than in control ovaries. We identified 13 regions with a high mean amount of 23- to 29-nt RNAs (MA plot red dots in Fig. 3A), including *white* (*w*) sequences (R100) corresponding to *BX2* [*P(lacW)* transgenes] and *jing* (R4), the gene flanking the *42AB* locus, the largest natural germline piRNA cluster in *Drosophila melanogaster*. Other selected regions contain genes mostly coding for transcriptional factors implicated in embryonic development (Fig. 3A). These results were confirmed by a sRNA sequencing (sRNA-seq) DESeq2 analysis focusing on *Drosophila* genes (r6.36). This independent approach confirmed that the genes embedded in the additional piRNA-producing regions produce significantly more piRNAs in *Kdm3* GLKD than in control (fig. S5, A and B). sRNAs produced by these regions originate from both strands (Fig. 3B), the 23- to 29-nt RNAs present an enrichment in U at 5′ end (63.5% 1U, *n* = 98,560) and A at the 10th position among the paired reads (56% 10A, *n* = 1397; Fig. 3C, left). The calculation of the 5′ overlap probability *Z* score confirmed the presence of a ping-pong signature for these 23- to 29-nt RNAs (Fig. 3C, right). It was shown that the Rhino machinery enables promoter-independent dual-strand transcription and suppresses splicing from piRNA cluster transcripts (*9*, *11*). As intron-containing genes are present in the 123 regions, we compared the number of 23- to 29-nt reads per kilobase originating from introns and exons. Comparable rates were observed [introns 2.39 RPKM (per kb and per million of reads) versus exons 1.98 RPKM], suggesting that transcripts from these regions are not spliced before sRNA production (table S5). Higher values, but still comparable between introns and exons, were obtained when considering only the 13 strongest 23- to 29-nt-producing regions (introns 4.46 RPKM versus exons 3.49 RPKM; table S5). Together, these data strongly suggest that these 23- to 29-nt RNAs are bona fide piRNAs.

RNA-seq experiments performed on total ovarian RNAs followed by a DESeq2 analysis between *Kdm3* GLKD and control revealed that among the 17,612 genes of *D. melanogaster*, 1283 are significantly up-regulated (7.3%) and 1399 are down-regulated (7.9%, see also MA and volcano plots on fig. S5, C and D). Among the 275 genes embedded in the 123 regions producing de novo piRNAs, 112 genes are significantly up-regulated (40.7%) and 31 genes are down-regulated (11.3%; fig. S5, C and D). Many genes embedded in those regions simultaneously show an increase in RNA steady-state level and a de novo piRNA production (Fig. 3D). Notably, in wild-type condition, numerous genes contained in these regions are not transcribed in ovaries. RT-qPCR experiments confirmed increased RNA levels in *Kdm3* GLKD context and further showed that they depend on the transcription factor Moonshiner (Moon) (Fig. 3E; see statistical analysis in table S6). Moon has been characterized as part of a Rhino-dependent transcription machinery that enables the initiation of germline piRNA clusters transcription (*11*). Together, these results strongly support that these regions have become genuine double-strand piRNA clusters.

### Chromatin modifications can initiate piRNA production in ovaries

**Fig. 4.**
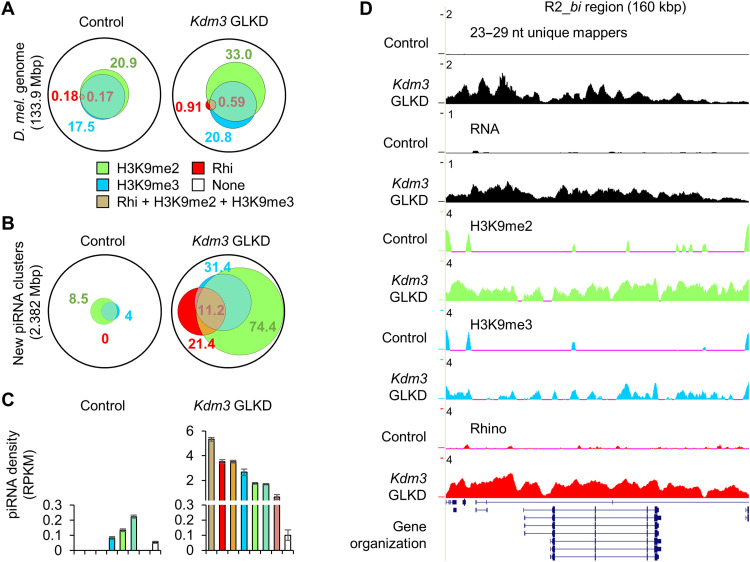
De novo piRNA production is associated with chromatin modifications. (**A**) Venn diagram of ChIP-seq analyses revealing genome proportions that are enriched in H3K9me2, H3K9me3 and/or Rhino in control or *Kdm3* GLKD. (**B**) Venn diagram showing the proportion of H3K9me2, H3K9me3, and/or Rhino enrichments upon the 2.382 Mb covered by the additional piRNA clusters (as defined in Fig. 3A) in control and in *Kdm3* GLKD. (**C**) Density of 23- to 29-nt unique mappers (in RPKM) by the 123 regions according to their chromatin state in control and in *Kdm3* GLKD. (**D**) Summary maps of small RNA sequencing (sRNA-seq) (23 to 29 nt) unique mappers, RNA seq, ChIP-seqs on R2_*bi* region. *Y* axis values are in rpm.

We therefore determined H3K9me2, H3K9me3, and Rhino (Rhi) occupancy through chromatin immunoprecipitation followed by sequencing (ChIP-seq) from control and *Kdm3* GLKD ovaries. MACS2 analyses revealed that in control ovaries, 19.5% of the genome is enriched in H3K9me2 and 16.3% in H3K9me3, which almost completely overlap H3K9me2-enriched regions (97.5% of H3K9me3-enriched regions are also H3K9me2 enriched; Fig. 4A and table S7). Rhi-enriched regions represent only 0.17% of the genome (~244 kb), a proportion compatible with previous observations (Fig. 4A and table S7) (*6*). A total of 0.16% of the genome is co-enriched in Rhi, H3K9me2, and H3K9me3, meaning that 93.3% of the Rhi-enriched regions are co-enriched with H3K9me2 and H3K9me3 (Fig. 4A and table S7). This strong interlocking suggests a functional relationship between the H3K9 methylation level and Rhi. In *Kdm3* GLKD ovaries, 30.7% of the genome is enriched in H3K9me2, likely as the direct consequence of the Kdm3 H3K9 demethylase depletion (Fig. 4A). This increase in H3K9me2 affects also the H3K9me3 distribution since a larger part of the genome is now enriched in H3K9me3 (19.3%), but the proportion of overlapping with H3K9me2-enriched regions remains stable (Fig. 4A and table S7). More spectacularly, the genome proportion on which Rhi binds is five times greater in *Kdm3* GLKD (0.85% versus 0.17%). A total of 0.55% of the genome (~790 kb) became co-enriched in Rhi, H3K9me2, and H3K9me3, representing 63.5% of Rhino-enriched regions (Fig. 4A and table S7). Considering the 2382 kb of sequences corresponding to the additional piRNA clusters determined through differential production of 23 to 29 nt in *Kdm3* GLKD versus control (Fig. 3A), we noticed that in the control context, they were poorly enriched in H3K9me2 (8.5%) or H3K9me3 (4%) and that none of them were enriched in Rhi (Fig. 4B and table S8). In *Kdm3* GLKD background, the chromatin landscape of these regions is drastically modified since 74.4% of the sequences were enriched in H3K9me2, 31.4% in H3K9me3 and 21.4% in Rhi (Fig. 4B). The proportion of sequences enriched in all of the three marks reached 11.2% (~268 kb). Among the 123 piRNA-producing regions, 69 present at least one Rhino peak enrichment. These 69 clusters totalize 1.855 kb length, thus representing 77.9% of the total size of the additional regions, and produce 93.2% of the de novo piRNAs. This result suggests that most of the additional piRNA production is Rhino dependent. For other regions, we cannot exclude Rhino-independent mechanisms or a noise effect (false positives) due to our analyses and used thresholds.

Last, we calculated the piRNA density for each subtype of enrichment and showed that the density is higher for regions that are co-enriched with Rhi, H3K9me2, and H3K9me3 (5.34 piRNA RPKM; Fig. 4C and table S9). This result highlights the crucial relationship between these chromatin modifications and the piRNA production. Genomic analyses were summarized and illustrated for the R2_*bi* region (Fig. 4D) and other regions (fig. S6). The 11 regions that showed less piRNA production in *Kdm3* GLKD context present also a decrease of Rhino binding (fig. S7). We thus propose that these regions correspond to endogenous piRNA clusters in control flies but that in *Kdm3* GLKD context, Rhino could be relocated from these regions to the additional producing ones.

Together, our results show that Kdm3 counteracts piRNA cluster determination and piRNA production from several specific gene-containing regions, thus revealing that a molecular control based on chromatin state rather than a by-default state exists to maintain some CDSs in a piRNA nonproducing state.

### Inheritance of auto-immune piRNAs may cause developmental defects in the offspring

**Fig. 5.**
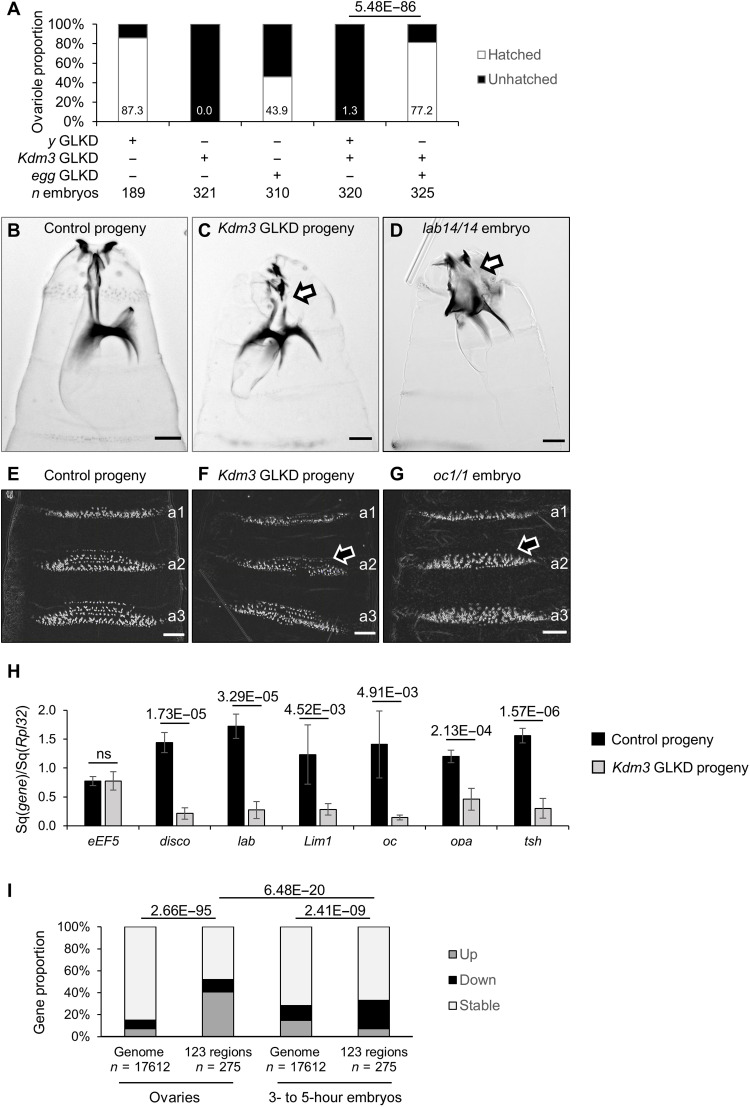
The progeny of *Kdm3* GLKD females display strong maternal phenotypes. (**A**) As indicated above each column in percentage, the hatching rate of the *Kdm3* GLKD progeny is strongly impaired when compared to control (*y* GLKD). Double *Kdm3* and *egg* GLKD almost fully rescues the hatching rate. (**B** and **C**) Sclerotized structures of the head of unhatched embryos issued from control females (B) or *Kdm3* GLKD females (C). The lack of continuity (as shown by an arrow) between the sclerites in the anterior part and the more posterior structures (ventral plate and arms) is even stronger in a *labial* complete loss-of-function embryo (homozygous for *lab*^14^) (**D**). Scale bars, 30 μm. (**E** to **G**) Ventral cuticular pattern of the embryos (abdominal a1 to a3 denticle bands) reveals the loss of denticle rows in the anterior region of the abdomen (as shown by an arrow) in the *Kdm3* GLKD progeny (F) compared to control (E). This phenotype is reminiscent of *oc/otd* loss of function (G). (**H**) RT-qPCR measurements of transcripts in embryos arising from *Kdm3* GLKD females using specific primers corresponding to tested genes and *Rpl32* primers as reference. They reveal that up-regulated genes embedded into deregulated regions in the mothers’ ovaries (Fig. 3) are down-regulated in their progeny. Number of biological replicates is *n* = 4 for all samples except for *disco*, *lab*, and *opa* control (*n* = 5) and *Lim1* and *tsh* control (*n* = 6). Error bars are SD. *P* values are indicated over the bars (bilateral *t* test). (**I**) Comparison of gene expressions that are overexpressed (up), down-regulated (down), or stable in *Kdm3* GLKD versus control in ovaries or in 3- to 5-hour embryos. Proportions were measured from DESeq2 analyses performed on corresponding RNA-seq data. *n*, gene number. *P* value is from Pearson’s χ^2^ test.

We then addressed whether these unexpected ovarian piRNAs might affect offspring. The hatching rate of the embryos laid by *Kdm3* GLKD females was severely impaired, whatever their genotype, thus revealing a strong maternal effect (Fig. 5A). A double GLKD of *Kdm3* and *egg* in females markedly increased the hatching rate of their progeny (Fig. 5A). This result reinforces the hypothesis that the developmental issues of the embryos laid by the *Kdm3* GLKD females are linked to the emergence of the additional piRNA-producing regions. Embryos issued from *Kdm3* GLKD females stopped their development at different stages, 17% (*n* = 369) reaching the first larval stage (L1) without hatching. The analysis of their cuticular phenotypes revealed defects in the mouth formation (Fig. 5C) and in the denticle belts (Fig. 5F) when compared to controls (Fig. 5, B and E). These phenotypes were reminiscent of *labial* (*lab*) and *occeliless* (*oc*, previously known as *orthodenticle*, *otd*) loss of function, respectively (Fig. 5, D and G) (*42*, *43*). *lab* and *oc* are included in the additional piRNA-producing regions defined in *Kdm3* GLKD ovaries (Fig. 3). RT-qPCR experiments performed on 3 to 5 hours embryos revealed that *lab* and *oc*, as well as most of the genes embedded in these additional piRNA clusters were less expressed in *Kdm3* GLKD female progeny when compared to control progeny (Fig. 5H). RNA-seq experiments of embryos coming from *Kdm3* GLKD or *w* GLKD females (fig. S8 and table S10) revealed that 40.7% of the genes belonging to the 123 regions are overexpressed in *Kdm3* GLKD ovaries compared to 7.3% in embryos, while 11.3% of the genes are underexpressed in ovaries compared to 25.8% in embryos (Fig. 5I). Together, these data suggest that de novo piRNAs are produced in the ovaries of *Kdm3* GLKD females from a modified transcription induced by chromatin changes and that their inheritance to progeny could impair the expression of several corresponding embryonic developmental genes.

**Fig. 6.**
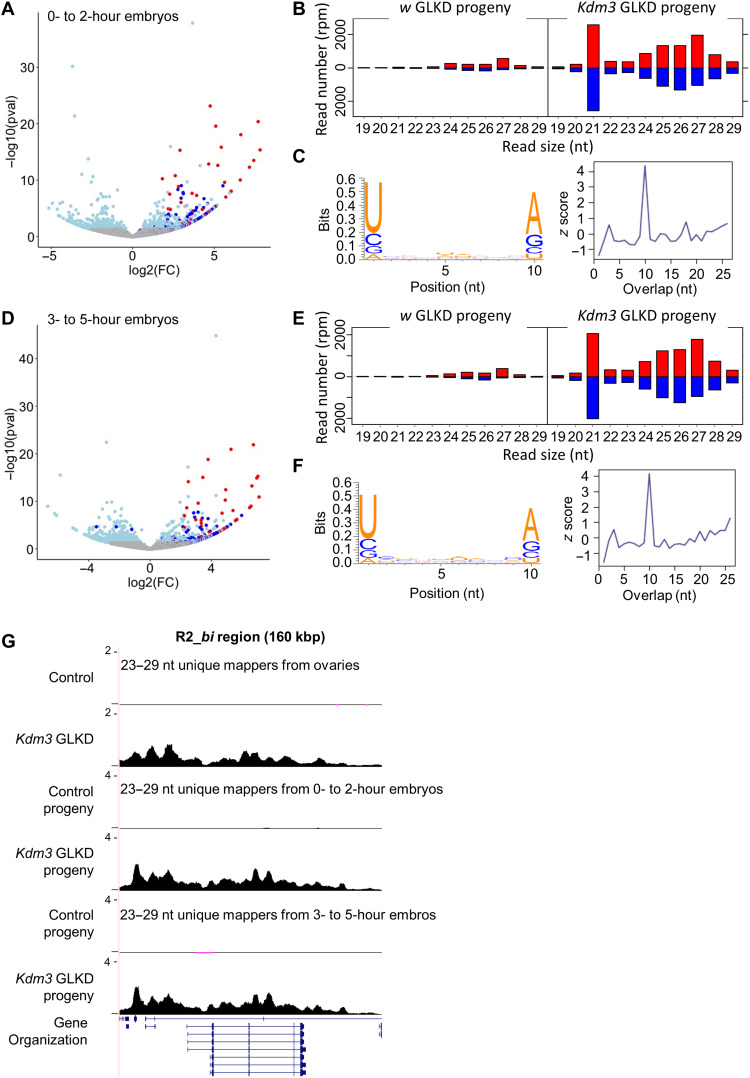
Inheritance and stability of auto-immune piRNAs. (**A**) Volcano plot showing the differential detection of 23- to 29-nt RNA matching genes in *Kdm3* GLKD progeny (0- to 2-hour embryos) compared to *w* GLKD progeny (as control). Red dots correspond to genes contained in the 13 most producing piRNA regions in *Kdm3* GLKD ovaries (same as Fig. 3A), dark blue dots are genes contained in the 123 additional producing regions, light blue dots are genes presenting a significant difference (*P* value <0.05), and gray dots are nonsignificant differentially expressed genes (*P* value >0.05). (**B**) Comparison of the size distribution of small RNAs (sRNAs) (19 to 29 nt) matching the 13 major producing regions [red dots in (A)] between control and *Kdm3* GLKD progenies (0- to 2-hour embryos). Positive and negative values correspond to sense (red) and antisense (blue) reads, respectively. (**C**) Logo showing the enrichment in U1 and A10 of the 10-nt paired sRNAs that match the 13 major producing regions in *Kdm3* GLKD and their 5′ overlap probability *z* score. (**D**) Same as (A), except that 23- to 29-nt RNAs were extracted from 3- to 5-hour embryos. (**E** and **F**) Same as (B) and (C), except that it refers to sRNAs from 3- to 5-hour embryos. (**G**) Summary maps of sRNA-seq (23 to 29 nt) unique mappers from control or *Kdm3* GLKD ovaries or from their respective progeny, 0- to 2- or 3- to 5-hour embryos. *Y* axis values are in rpm.

To reinforce this hypothesis, we analyzed sRNA-seq from embryos (0 to 2 and 3 to 5 hours) coming from *Kdm3* GLKD females and from *w* GLKD females as control. RNAs (23 to 29 nt) homologous to the 123 regions that newly produce piRNAs were specifically detected in the *Kdm3* GLKD progeny (Fig. 6, A and D). As in ovaries (Fig. 3), they present all characteristics of genuine piRNAs (Fig. 6, B, C, E, and F) and a similar distribution profile (Fig. 6G; *bi* region as an example and other regions fig. S9). The presence and the stability of these ectopic piRNAs in the progeny strengthen the hypothesis of a negative role on embryonic development through their maternal inheritance. As piRNAs were often compared to a genomic immune system, we propose to name these unexpected piRNAs, “auto-immune piRNAs.”

## DISCUSSION

**Fig. 7.**
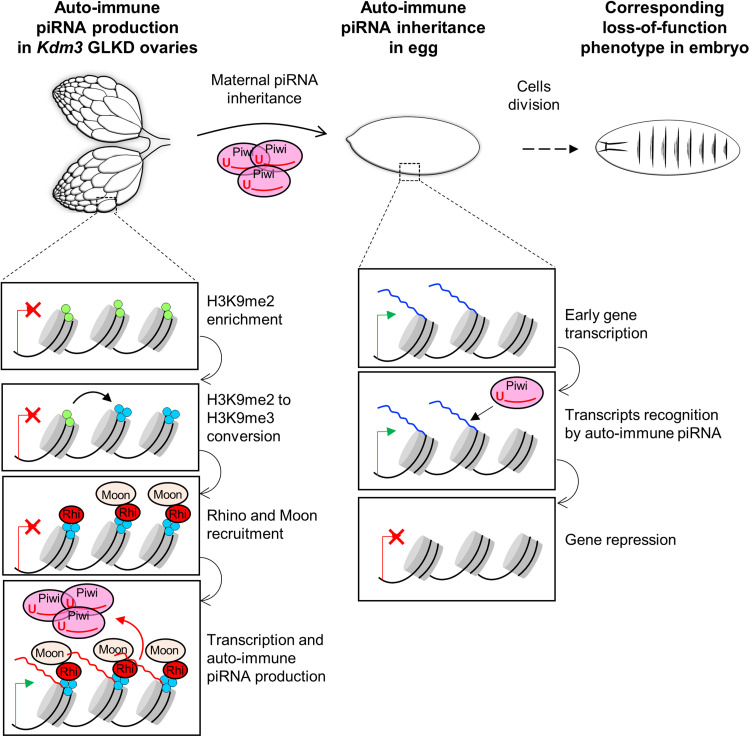
Working model for auto-immune piRNAs. This model summarizes how modification of chromatin marks in the *Kdm3* GLKD ovaries could lead to the production of additional piRNAs from gene-coding regions. Then, the maternal inheritance of these ectopic piRNAs could lead to embryonic developmental phenotypes mimicking a loss of function of the corresponding genes in the progeny.

In this study, we determined that *Kdm3* down-regulation increases H3K9me2 chromatin enrichment globally by 50% on the whole genome in the *Drosophila* ovary. Consequently, a subset of these regions were also found enriched in H3K9me3 probably due to the action of the histone methyltransferase Egg/SetDB1, shown to be required for H3K9 trimethylation in the ovaries (*44*, *45*). Among them, some gene-containing regions were also found enriched in Rhino, leading to the formation of de novo piRNA clusters associated with the production of auto-immune piRNAs in ovaries. These auto-immune piRNAs could be maternally inherited, thus leading to embryonic developmental defects (Fig. 7). When piRNA clusters are correctly defined, the epigenetic information is conserved to the next generation via maternal piRNA inheritance: piRNA clusters in the progeny are the same than those from mother (*6*, *13*, *14*). In this study, we showed that *Kdm3* GLKD leads to the emergence of de novo piRNA clusters even in absence of maternally inherited piRNA targeting these sequences. Since unexpected piRNAs are produced from these additional piRNA clusters, the epigenetic information that is transmitted to the next generation is modified. Mutation of *Suppressor of Hairy wing* [*Su(Hw*)], a protein that binds an insulator sequence contained in the *gypsy* retrotransposon, also leads to the production of de novo piRNAs from several gene-coding regions (*46*). *Su(Hw)* mutant female progeny is not viable like progeny of *Kdm3* GLKD female, suggesting that these de novo piRNAs could be functional. However, there is no overlap between the additional piRNA clusters seen in *Su(Hw)* mutant and *Kdm3* GLKD contexts, suggesting that different genomic regions and mechanisms are affected. Independent strategies could have been developed to ensure correct piRNA cluster determination and fix piRNA cluster borders. As a functional antagonist of egg/SetDB1, Kdm3 could thus be a guardian of piRNA cluster boundaries, whose role can be to limit the spreading of piRNA cluster loci by demethylating H3K9, thus avoiding Rhino from binding out of these borders. This hypothesis is well sustained by the *jing* locus, which is adjacent to the *42AB* cluster, the major germline piRNA cluster (*3*), and which becomes one of the additional piRNA clusters upon *Kdm3* GLKD. However, being at the border of existing piRNA clusters is not the only role of Kdm3 since a lot of additional piRNA regions are not in the vicinity of known piRNA clusters. Several studies propose that single TE insertions can become piRNA clusters (*47*–*49*) and one can imagine that Kdm3 blocks the extension of H3K9 methylation from single TE insertions. While we cannot totally exclude this possibility, we think that under this hypothesis, loss of Kdm3 would lead to piRNA production from the vicinity of a lot of single TE copies dispersed throughout the genome instead of revealing a small number of well-defined regions as shown here. Thus, we rather favor the hypothesis of another chromatin mark that specifies the recruitment of Rhino on these particular regions, together with the methylation state of H3K9. The transcription state of these regions could also be a key factor since a study points to the role of dual-transcription before the Rhino recruitment to become a piRNA cluster: Tethering Rhino to a transgenic sequence leads to the production of transgenic piRNAs only when the transgenic sequence is transcribed from both DNA strands thanks to divergent promoters (*9*). However, de novo piRNA-producing regions in *Kdm3* GLKD background were not transcribed in the wild-type ovaries, suggesting that dual-strand transcription is not always a prerequisite for becoming a piRNA cluster. From these regions, the transcription and the production of piRNA from both strands in *Kdm3* GLKD background is very likely a consequence of the recruitment of Rhino and its partners (*11*). Several parameters remained to be elucidated to understand what defines piRNA clusters. At each generation, the genome must equilibrate the balance between the protection of the genome against TE and appropriate developmental gene expression. This balance is dependent on tight control that defines genomic regions through chromatin modifications that regulate piRNA production in the female germline and then transmit it to the next generation. Our results highlight the importance of the chromatin state in piRNA cluster determination and show that maternal inheritance of homologous piRNA can be bypassed to produce piRNAs from new loci. Hence, a non–piRNA-producing state is therefore not a by-default state but rather a cellular lock that is actively controlled for several genomic loci.

## MATERIALS AND METHODS

### Transgenes and strains

All transgenes are in the *w*^1118^ background. The *P(lacW)* transgene (FBtp0000204) is composed of 5′ and 3′ *P* element extremities (*P5* and *P3*, respectively), *Escherichia coli lacZ* gene (*lacZ*), *D. melanogaster miniwhite* gene (*W*) and backbone plasmid pBR322 sequence (pBR). The *BX2* line (FBti0016766) carries seven *P(lacW)* transgenes, inserted in tandem and in the same orientation at cytological site 50C on the second chromosome (*50*). The transgene insertion site is located in an intron of the *AGO1* gene (*13*). Homozygous individuals are rare and sterile and the stock is maintained in heterozygous state with a *Cy*-marked balancer chromosome. β-Galactosidase activity from these transgenes is not detected in the germline. *P(lacZ)* corresponds to *BQ16* (FBti0003435) expressing *lacZ* only in the germline from *P(A92)* (FBtp0000154), a *P-lacZ-rosy* enhancer-trap transgene located on the third chromosome. Homozygous flies are viable. Another *P(lacZ)* located on the second chromosome at 60B7 was used, a *P(PZ)* transgene (FBtp0000210) corresponding to a *P-lacZ-rosy* enhancer-trap transgene and expressing β-galactosidase in the germline and somatic cells of the female gonads [Bloomington stock number *11039* (FBst0011039)]. Homozygous flies are not viable and the stock is maintained over a *Cy*-marked balancer chromosome. The *P-1152* line carries two *P(lArB)*, *P-lacZ-rosy* enhancer-trap transgenes (FBti0005700) that are inserted into subtelomeric sequences of the *X* chromosome (*51*). The *nosGAL4* transgene mainly used is from *w[1118]; P{w[+mC] = GAL4::VP16-nos.UTR}CG6325*[MVD1] line (FBti0012410). The *nosGAL4* from the w[*]; PBac{w[+mW.hs] = GreenEye.nosGAL4}Dmel6 line (FBti0131635) was also occasionally used and gave the same results. *Otu* alleles are *otu*^11^, an EMS induced C343Y substitution in the TUDOR domain and hence specifically affecting the activity of the 104-kDa Otu isoform (*52*) or *otu*^7^, an EMS induced nonsense mutation K424@ (or K382@ depending on the isoform), each leading to truncated nonfunctional proteins. *Kdm3* alleles are *Kdm3^KO^*, a gift of M. Buszczak (*30*) and *Kdm3^MI13382^*, a MiMic transgene inserted in the JmJc domain that inactivates the gene (Bloomington stock center #59126) (*34*). Small hairpin RNAs (shRNAs) are listed in table S1. *hfp* and *Kdm3* shRNA that allow *BX2* activation are TRiP line #34785 and TRiP line #32975, respectively. *Egg* shRNA showing epistasis interactions with *hfp* and *Kdm3* shRNA is TRiP line #36797. Mock shRNA are *y* shRNA TRiP line #64527 on chromosome 2 or *w* shRNA TRiP line #33644 on chromosome 3. Flies were grown on a corn-based medium made of agar-agar (11 g/liter), yeast powder (75 g/liter), corn flour (90 g/liter), and 35 mM methyl 4-hydroxybenzoate as antifungi. All crosses were performed at 25°C in incubators. Humidity was controlled through the use of desiccators. No special precautions were taken with regard to the light/dark cycles.

### Cloning procedures

*EGFP* sequences were amplified by PCR from *pGEM5Z(+)-EGFP* (Addgene #65206) and were cloned into *p(UASp)* (FBtp0010350) at the Bam HI site. *Kdm3* CDS was amplified from wild-type genomic DNA (*CantonS*) and cloned into *p(UASp-EGFP)* at the Eco RI site in frame with the *EGFP* sequence (for primers, see table S11). Sequence integrity was verified by sequencing. The sequence is available at Genbank under accession number OP765907. Plasmid *p(UASp-Kdm3-EGFP)* was then injected into *w*^1118^ embryos by BestGene Inc. Several independent lines were recovered. An insertion on the *X* chromosome was used for rescue experiments.

### β-Galactosidase staining

Ovarian *lacZ* expression assays were carried out using X-gal (5-bromo-4-chloro-3-indolyl-β-d-galactopyranoside) overnight staining at 37°C as previously described (*53*), except that ovaries were fixed afterward for 10 min. After mounting in glycerol/ethanol (50/50), ovarioles were counted as fully repressed if all egg chambers were white (100% repression), nonrepressed if all egg chambers were blue (0% repression) or mixed if some egg chambers were blue, while others were white (mixed repression). Images were acquired with an Axio-ApoTome (Zeiss) and ZEN2 software.

### Cuticle preparation

Experiment has been done as previously described (*54*). Laid embryos were allowed to age for 24 hours at 25°C. They were rinsed in water and then dechorionated using 8% sodium hypochlorite solution for 2 min. Embryos were rinsed again in water and the vitelline membrane was removed mechanically using a needle. Water was replaced by 1:1 lactic acid:Hoyer’s–based medium. Embryos were transferred onto a glass microscope slide and placed under a cover slip. Slides were incubated at 60°C overnight. Phase contrast images were obtained using the ×20 objective of an Axio-ApoTome (Zeiss) and ZEN2 software.

### RNA extraction

For ovaries samples, 10 to 20 pairs of ovaries were manually dissected in 1× phosphate-buffered saline (PBS). For embryo samples, 15 to 20 μl of embryos aged of 0 to 2 or 3 to 5 hours after egg laying were collected, washed with water and flash-frozen in liquid nitrogen. Total RNA was extracted using TRIzol (Life Technologies). For the RNA precipitation step, 100% ethanol was used instead of isopropanol and washes were made in 80% ethanol instead of 75% ethanol. Up to six biological replicates were used for each genotype. Exact number of biological replicates is indicated for each experiment.

### RT-qPCR experiments

For each sample, 2 μg of total RNA was treated with deoxyribonuclease (DNase; Fermentas). For RT-qPCR experiments, 1 μg of DNase-treated RNA was used for reverse transcription using random hexamer primers (Fermentas). RT-qPCR was performed on triplicates of each sample. *RpL32* was used as reference (for primers, see table S11). The same series of dilution of a mix of different RT preparations was used to normalize the quantity of transcripts in all RT preparations leading to standard quantity (Sq) values. Variations between technical triplicates were very low when compared to variations between biological replicates. The mean of the three technical replicates was then systematically used (meanSq). For each biological sample, we calculated the ratio meanSq(gene)/meanSq(*RpL32*) to normalize the transcript quantity. Then, the mean of each sample ratio was used to compare the two conditions.

### Native RNA-IP followed by RT-qPCR

For each sample, 80 pairs of ovaries from well-fed females (2 or 3 days) were manually dissected in cold 1× PBS, flash-frozen in liquid nitrogen and conserved at −80°C. Ovaries from *hfp-GFP* and *GFP*-negative control lines were lysed and grinded with pestle in RNA-IP lysis buffer [159 m M KCl, 25 mM tris (pH 7.4), 5 mM EDTA, 0.5 mM dithiothreitol (DTT), 0.5% Nonidet P-40], freshly supplemented with Protease Inhibitor Cocktail (one tablet per 10 ml of RNA-IP lysis buffer) and RNase inhibitor (40 U per 1 ml of RNA-IP buffer). Lysates were sonicated using Bioruptor (Bioruptor Standard Diagenode) for 7 min (15-s ON and 60-s OFF) and cleared by centrifugation at 12,000 rpm for 15 min at 4°C. Ten percent of cleared lysate was set aside to serve as input samples and the remainder was incubated at 4°C with anti-GFP antibodies (Roche #11814460001) for 3 hours under gentle rotation. Magnetic beads coupled to G protein (Dynabeads #10003D, Invitrogen) were washed two times with RNA-IP lysis buffer, transferred into immunoprecipitated lysate, and incubated at 4°C for 1 hour under gentle rotation. The beads were washed five times with RNA-IP lysis buffer for 10 min under gentle rotation at 4°C. Total RNA was extracted from input and beads using TRI reagent (Sigma-Aldrich catalog no. T9424) as described in reagent manual (www.sigmaaldrich.com/technical-documents/protocols/biology/tri-reagent.html). For RNA precipitation step, 100% ethanol was used instead of isopropanol and for wash steps 80% ethanol was used instead of 75% ethanol. For each genotype, three to four biological replicates were used. For each input sample, 2 μg of total RNA was treated with DNase (New England Biolabs) and for the RT-qPCR experiment, 1 μg of DNase-treated RNA was used for reverse transcription using random hexamer primers (Fermentas). For each immunoprecipitated sample, total RNA was treated with DNase, and total DNase-treated RNA was used for reverse transcription using random hexamer primers. Real-time PCR was performed on duplicates for each biological sample leading to cycle threshold (Ct) values (for primers, see table S11). Variations between technical duplicates were very low compared to variations between biological replicates. The mean of the two technical replicates was then systematically used (meanCt). Data have been analyzed as described in (*55*). For each sample, the IP fraction is normalized beside input to take account of sample preparation difference as follows: ΔCt [normalized RIP] = (meanCt [RIP] − {meanCt [Input] – Log2 (input dilution factor)}) where meanCt [RIP] is the Ct value measure for immunoprecipitated samples, meanCt [Input] is the Ct value measure for input and input dilution factor corresponds to the RNA fraction set aside for input (in this experiment, 10% of RNA fraction was set aside, thus input dilution factor is 10). Antibody signal specificity was confirmed by comparing *hfp-GFP* to *GFP*-negative control, fold enrichment was calculated for each sample as follows: fold enrichment = 2^(−ΔCt[normalized RIP]−ΔCt[normalized NS])^.

### sRNA-seq analyses

AsRNA fraction of 15 to 30 nt in length was obtained following separation of total RNA extracted from dissected ovaries or embryos on a denaturing polyacrylamide gel. This fraction was used to generate multiplexed libraries with Illumina TruSeq Small RNA library preparation kits (RS-200-0012, RS200-0024, RS-200-036, or RS-200-048) at Fasteris (www.fasteris.com). 2*S* RNA (30 nt) contamination was reduced in the final library by blocking the 5′ adapter ligation to the 2*S* RNA fragments using a 2*S* rRNA complementary RNA oligonucleotide. Libraries were sequenced using Illumina HiSeq 2000 and 2500. Sequence reads in fastq format were trimmed from the adapter sequence 5′-TGGAATTCTCGGGTGCCAAG-3′ and matched to the *D. melanogaster* genome release 6 (dm6) using Bowtie (Table 1 and table S2) (*56*). For subsequent analyses, we used a cleaned version of the genome in which Y, U, and mitochondrial chromosomes were removed. This version, named “dm6_clean”, has been deposited at the Gene Expression Omnibus (GEO) under accession number GSE203279.

Sequence length distributions, sRNA mapping, and sRNA overlap signatures were generated from bowtie alignments using Python and R (www.r-project.org/) scripts, which were wrapped and run in Galaxy instance publicly from ARTbio platform available at http://mississippi.fr (see also Supplementary Data). Tools and workflows used in this study may be downloaded from this Galaxy instance. For library comparisons, read counts were normalized to 1 million reads (table S2). For sRNA mapping (Figs. 1 and 3 and fig. S2), we took into account only 23- to 29-nt RNA reads that uniquely aligned to reference sequences (unique mappers). Logos were calculated using Weblogo (*57*) from 3′ trimmed reads (23 nt long) matching *P(lacW)* (Fig. 1, L and M) or selected genomic regions (Fig. 3C). The percentage of reads containing a “U” at the first position was calculated with all 23- to 29-nt RNA matching the reference sequence. Distributions of piRNA overlaps were computed as first described in (*8*) and detailed in (*58*). Thus, for each sequencing dataset, we collected all of the 23- to 29-nt RNA reads matching *P(lacW)* whose 5′ ends overlapped with another 23- to 29-nt RNA read on the opposite strand. Then, for each possible overlap of 1 to 29 nt, the number of read pairs was counted. The percentage of reads containing an “A” at the 10th position was calculated within the paired 23- to 29-nt RNA matching the reference sequence as described in (*13*). sRNA sequences and projects have been deposited at the GEO under accession number GSE203279.

### RNA-seq analyses

Total RNA was extracted with TRIzol from hand dissected ovaries or 50 μl of 3- to 5-hours embryos in 1× PBS and 1 μg was treated with DNase (Fermentas). After an initial quality control, libraries were prepared using the RNA RiboZero Stranded protocol by Fasteris. Indexed adapters were ligated and multiplexed sequencing was performed using Illumina HiSeq 2000 and 2500 (table S2). A DESeq2 analyses on all *D. mel.* genes (release 6.36) was performed using the three biological replicates for each genotype, control, or *Kdm3* GLKD in ovaries and the two biological replicates in embryos. The original files have been deposited at the GEO under accession number GSE203279.

### ChIP-seq analyses

ChIP was performed as previously described (*59*) with minor modifications. In brief, 100 ovary pairs were manually dissected into Schneider media and cross-linked in 1% formaldehyde/PBS for 10 min at room temperature with agitation. The cross-linking reaction was quenched by STOP buffer (1× PBS, 0.1% Triton X-100, and 1 M glycine) and ovaries were washed in PBS and homogenized in a glass douncer: first slightly dounced in 0.1% PBST and centrifugated 1 min 400*g*, followed by strong douncing in cell lysis buffer buffer (85 mM KCL, 5 mM HEPES, 0.5% NP-40, 10 mM sodium butyrate, and EDTA free protease inhibitor cocktail; Sigma-Aldrich) following 5-min centrifugation at 2000*g*. We performed two washes with cell lysis buffer. The homogenates were then lysed on ice for 30 min in nucleus lysis buffer (50 mM HEPES, 10 mM EDTA, 0.5% *N*-lauryl sarkosyl, 10 mM sodium butyrate, and EDTA-free protease inhibitor cocktail; Sigma-Aldrich). DNA was sheared using a Bioruptor pico from Diagenode for 10 cycles (30-s on and 30-s off). The sonicated lysates were cleared by centrifugation and then incubated overnight at 4°C with anti-Rhino antibody. Then, 40 μl of Protein A Dynabeads was then added and allowed to bind antibody complexes by incubation for 1 hour at 4°C. For H3K9me2 and me3 ChIP, 50 μl of Protein A Dynabeads was first coated with the anti-H3K9me3 or me2 antibodies and then incubated with the chromatin overnight at 4°C. Following four washing steps with high-salt buffer [50 mM tris (pH 7.5), 500 mM NaCl, 0.25% Triton X-100, 0.5% NP-40, 0.5% bovine serum albumin, and 5 mM EDTA (pH 7.5)], DNA-protein complexes were eluted and de-crosslinked 10 hours at 65°C. RNA and protein was digested by RNase A and proteinase K treatments, respectively, before purification using phenol/chloroform protocol. Barcoded libraries were prepared using Illumina technology, which were sequenced on a NextSeq High (Illumina) by Fasteris for the ChIP H3K9me3 and H3K9me2 and by the Jean Perrin facility for the ChIP Rhino (table S7). Sequences and projects have been deposited at the GEO under accession number GSE203279.

### Data analyses

All analysis scripts were deposited on Dryad (https://doi.org/10.5061/dryad.n2z34tn1p). For alignments, we used a cleaned version of r6.36 version of *D. melanogaster* genome (dm6, Flybase) in which sequences corresponding to unmapped (chrU), Y chromosome (chrY), and mitochondrial chromosome (chrM) have been removed (dm6_clean).

For sRNA-seq analyses, we first discarded the reads matching tRNA (dmel-all-tRNA-r6.30, Flybase), miRNA (dmel-all-miRNA-r6.30, Flybase), miscRNA (dmel-all-miscRNA-r6.30, Flybase), and rRNA (dm3-rRNA-sequences, Galaxy Tutorial) using sR_Bowtie -m3 (Galaxy version 2.1.1). Then, unique mapper reads were obtained using sR_Bowtie -m0 (Galaxy version 2.1.1) and the dm6_clean genome as reference. Reads (23 to 29 nt) were aligned on 124 additional piRNA production loci with sR_Bowtie -m0 (Galaxy version 2.1.1). The reads were counted with Bamparse (Galaxy version 3.0.0). Then, the counts were normalized in rpm, and means and ratio were calculated to plot the log2FC (*Kdm3* GLKD compared to control) on the BaseMean.

For RNA-seq analyses, reads were aligned to genes’ reference genome (all-gene-r6.36.fasta, Flybase) with Bowtie_wrappers (Galaxy version 1.2.0) and counted with featureCounts (Galaxy version 1.6.4). The differential expression (*Kdm3* GLKD compared to control) was determined with DESeq2. The same procedure was used to identify genes with a differential production of piRNA except that the alignment was made with sR_Bowtie -m0.

For ChIP-seq analyses, reads were mapped on dm6_clean reference genome with BWA (Galaxy version 0.7.17.4). PCR duplicates were discarded using Picard tool (Galaxy version 2.18.2.1). Peak calling was made on mapped reads with MACS2 (Galaxy version 2.1.1.20160309.6) using following parameters: --gsize ‘120000000’ --keep-dup ‘1’ --qvalue ‘0.05’ --nomodel --extsize ‘200’ --shift ‘0’. Narrowpeak was used for libraries obtained with Rhino antibody and broadpeak calling was used for libraries obtained with H3K9me2 and H3K9me3 antibodies (--broad --broadcutoff = ‘0.1’). To define the whole enrichment, the ratio between the IP sample and input sample was calculated. A differential analysis was also performed by calculating the ratio between IP *Kdm3* GLKD samples and IP control samples. Overlapping enriched regions were determined using bedtools intersect intervals (bedtools Galaxy version 2.30.0). Overlaps between chromatin-marks–enriched regions and additional piRNA-producing regions were determined similarly. DNA sequences (in fasta format) from these overlaps were recovered from coordinates with GetFastaBed (bedtools Galaxy version 2.30.0). Unique mappers were aligned on these sequences using sR_Bowtie -m0 to determine the piRNA density.

UCSC views were made with BamCoverage (Deeptools Galaxy Version 3.3.2.0.0) for small- and long-RNA-seq. Bin size was 29 bp for small seq and 50 pb for RNA-seq and reads were normalized in rpm. For ChIP-seq analyses, BamCompare (Deeptools Galaxy version 3.3.2.0.0) was used with a bin size of 100 pb and the computation of the difference of the IP input reads in rpm.

## Notes

View/request a protocol for this paper from *Bio-protocol*.
