# Supplementary material for: The histone demethylase Kdm3 prevents auto-immune piRNAs production in Drosophila
Source: Sci Adv. 2023 Apr 7;9(14):eade3872. doi: 10.1126/sciadv.ade3872 (PMC10081847; doi:10.1126/sciadv.ade3872)
Supplement: Supplementary file 1 — Figs. S1 to S9 Tables S1 to S11 [file sciadv.ade3872_sm.pdf]

Science Advances
AAAS

Supplementary Materials for

The histone demethylase Kdm3 prevents auto-immune piRNAs production in *Drosophila*

Karine Casier *et al.*

Corresponding author: Antoine Boivin, antoine.boivin@sorbonne-universite.fr;
Laure Teysset, laure.teysset@sorbonne-universite.fr; Clément Carré, clement.carre@sorbonne-universite.fr

Sci. Adv. **9**, eade3872 (2023)
DOI: 10.1126/sciadv.ade3872

This PDF file includes:

Figs. S1 to S9
Tables S1 to S11

SUPPLEMENTARY MATERIALS

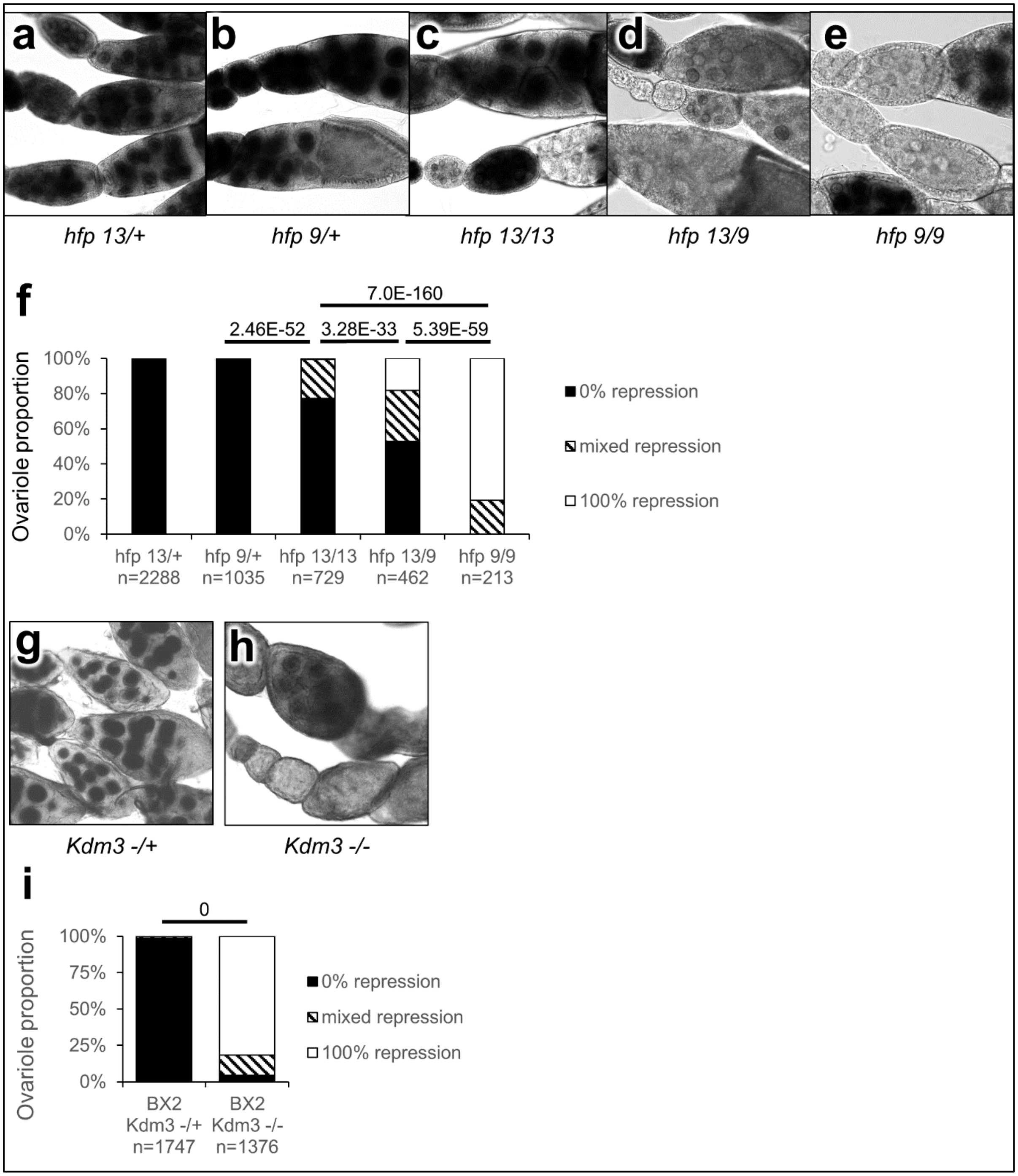

Figure S1. *hfp* and *Kdm3* mutation leads to the *de novo* activation of the *BX2* piRNA cluster. a-e, X-gal staining of ovaries. Genotypes are given. hfp^9 and hfp^{13} are two hypomorphic alleles allowing adult survival *(33)*. **f,** Ovariole repression was measured by counting the number of ovarioles showing no, mixed or complete repression of the *P(lacZ)* target among egg chambers. Interestingly, we observed that the

strongest hypomorphic allele (*hfp*^{9}) leads to the strongest *BX2* activation (almost 100% in homozygous state) while the weakest allele (*hfp*^{13}) leads to a weakest *BX2* activation. Heterozygous combination leads to an intermediate *BX2* activation. p-value are indicated above corresponding bars (Pearson's Chi-squared test). n = number of counted ovarioles. **g-h,** X-gal staining of ovaries. Genotypes indicated correspond to allelic combination of *Kdm3* loss-of-function alleles: a heterozygous *Kdm3*$^{KO/+}$ does not lead to the activation of *BX2* (**g**) while a *trans*-heterozygous loss-of-function combination leads to *BX2* conversion (**h**). **i,** Ovariole repression was measured as previously. *p*-value is indicated above corresponding bars (Pearson's Chi-squared test). n = number of counted ovarioles.

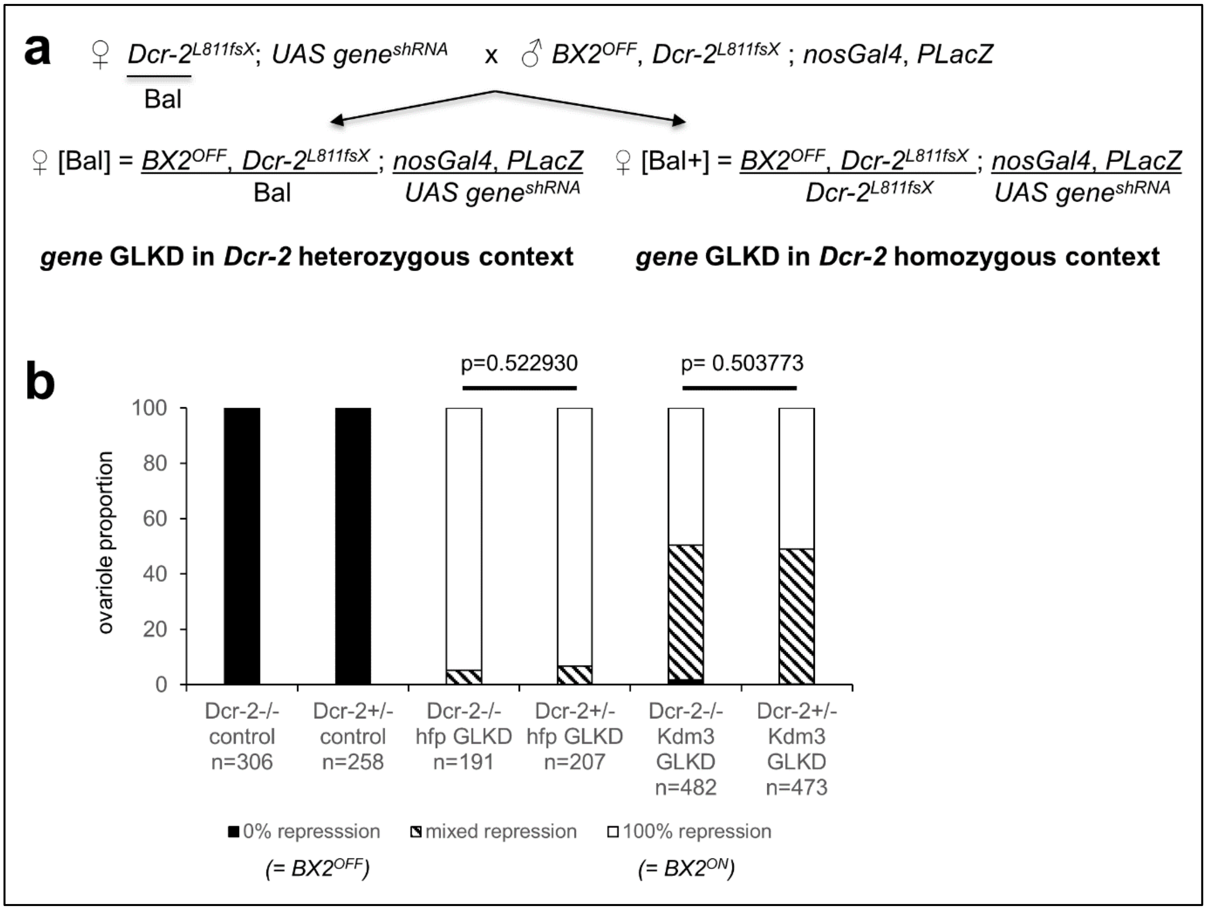

Figure S2. ***Dcr-2* mutant context does not impair activation of the *BX2* piRNA cluster in *hfp* and *Kdm3* GLKD. a,** Cross scheme performed. **b,** Ovariole repression was measured by counting the number of ovarioles showing no (0%), mixed or complete (100%) repression of the *P(lacZ)* target among egg chambers. *p*-values are indicated above corresponding bars (Kruskal-Wallis rank sum test). n = number of counted ovarioles.

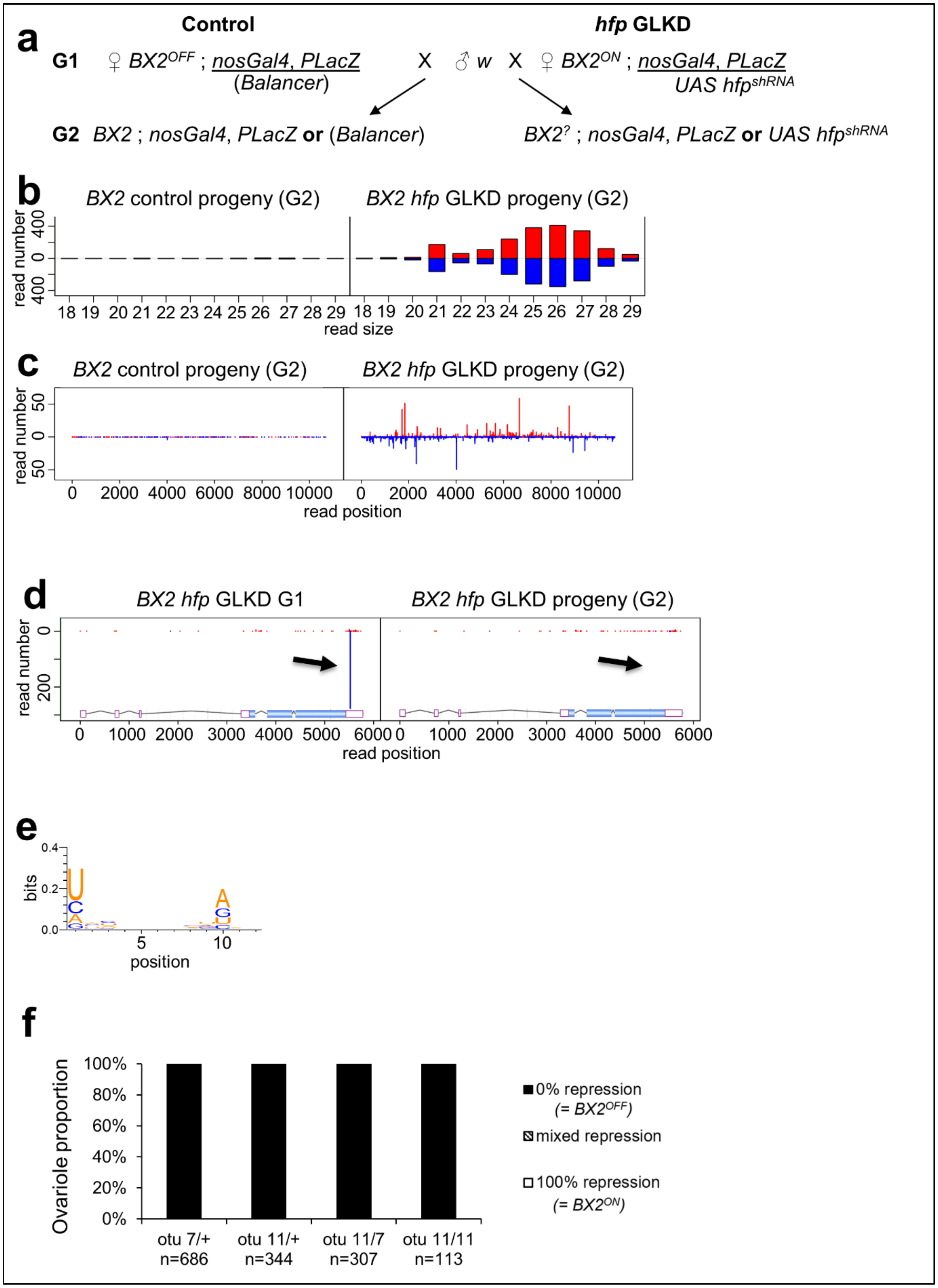
a
Control
hfp GLKD
G1 ♀ BX2OFF ; nosGal4, PLacZ / (Balancer) X ♂ w X ♀ BX2ON ; nosGal4, PLacZ / UAS hfpshRNA
G2 BX2 ; nosGal4, PLacZ or (Balancer)
BX2? ; nosGal4, PLacZ or UAS hfpshRNA
b
BX2 control progeny (G2)
BX2 hfp GLKD progeny (G2)
read number
read size
c
BX2 control progeny (G2)
BX2 hfp GLKD progeny (G2)
read number
read position
d
BX2 hfp GLKD G1
BX2 hfp GLKD progeny (G2)
read number
read position
e
bits
position
f
Ovariole proportion
otu 7/+ n=686
otu 11/+ n=344
otu 11/7 n=307
otu 11/11 n=113
0% repression (= BX2OFF)
mixed repression
100% repression (= BX2ON)

Figure S3. Stability of the *hfp* GLKD mediated *BX2* conversion at the next generation. a, Genetic crosses performed to analyze the progeny of control or *hfp* GLKD females. **b**, Comparison of the size distribution of ovarian small RNAs (18-29 nt) matching *BX2* sequences between progeny of control and *hfp* GLKD. Positive and negative values correspond to sense (red) and antisense (blue) reads, respectively. **c**, Comparison of unique 23-29 nt mappers along the *BX2* sequence between progeny of control and *hfp* GLKD. **d**, Comparison of multiple 18-29 mappers along the *hfp* sequence between *hfp* GLKD flies (G1) and their progeny (G2) shows that G2 flies no longer produce shRNA against *hfp* 3' UTR (as indicated by the black arrow). **e**, Logo showing the enrichment in U1 and A10 of the 10 nt paired small RNAs that match *BX2* in the *hfp* GLKD progeny. These piRNAs are enriched in U at position 1 (67.7% n=34609 reads) and exhibit a ping-pong signature as an enrichment of 10A among paired reads (47.4% n=2421). **f,** Combinations of *otu* mutant alleles do not induce *BX2* conversion. The parental cross corresponds to otu^{11} */ M5; P(lacZ)* females crossed by otu^{pat}; $BX2^{OFF}$ */ CyO* males. otu^{pat} can be otu^{11}, an EMS induced C343Y substitution in the TUDOR domain and hence specifically affecting the activity of the 104 kDa Otu isoform *(52)*, or otu^{7}, an EMS induced nonsense mutation K424@ (or K382@ depending on the isoform), leading to truncated non-functional proteins. Whatever the allelic combination, *BX2* never switched on.

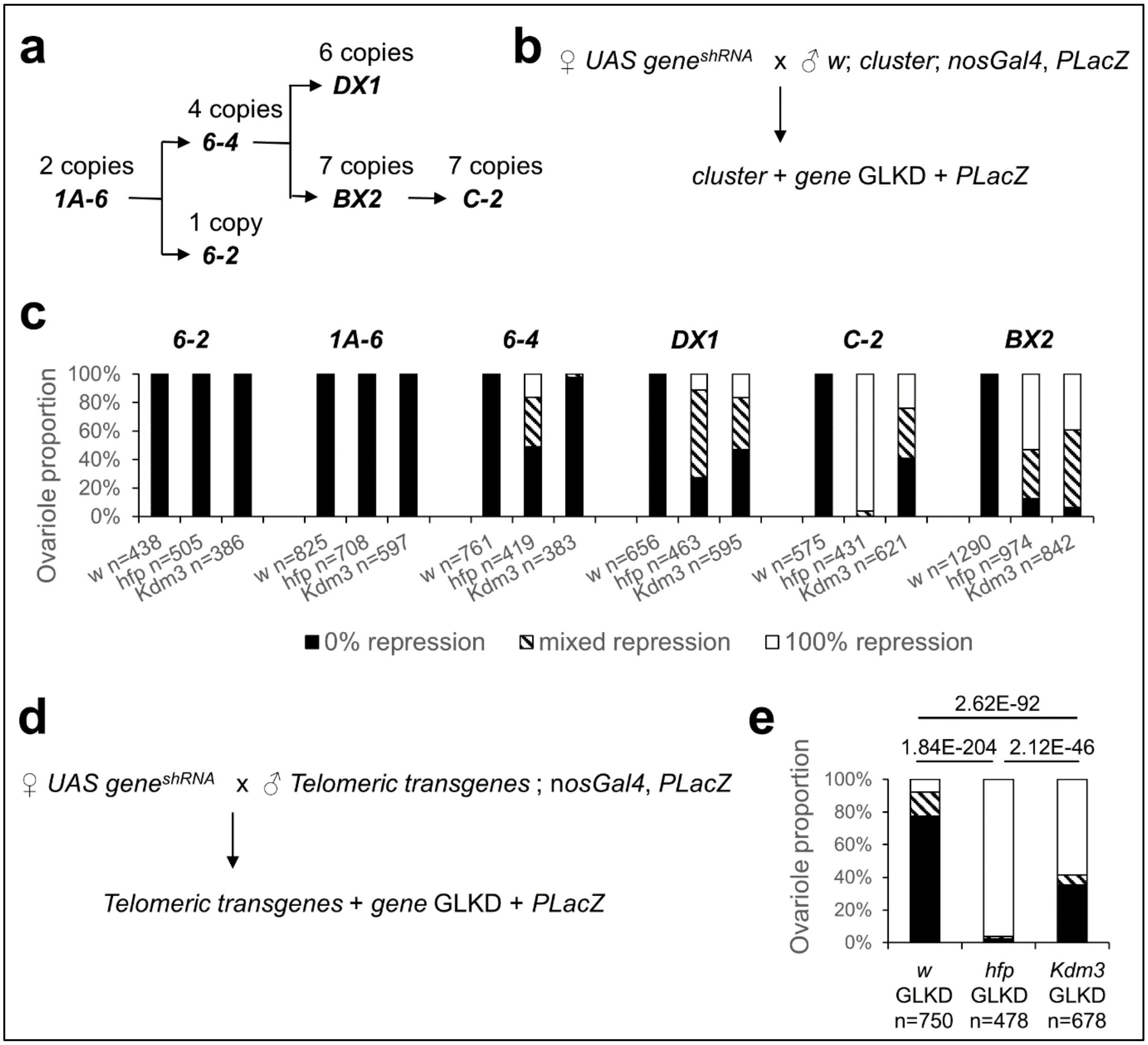

Figure S4. Other allelic piRNA clusters are activated upon *Kdm3* GLKD. a, Origin and number of *P(lacW)* copies in each studied cluster *(13,50,60)* . All clusters are inserted at the same locus. **b**, Each cluster was paternally inherited in order to measure its conversion rate in the ovaries of the progeny. The conversion was measured as the ability of the cluster to silence the *P(lacZ)* transgene. **c,** Histogram showing the conversion rate of the six clusters in three genetic backgrounds, *white (w)* GLKD serving as control, *hfp* GLKD or *Kdm3* GLKD. n = number of counted ovarioles. **d,** Following a similar scheme, telomeric transgenes (*P-1152*) were paternally inherited and their conversion rate was analyzed in the ovaries in the progeny as in **c**. **e,** Histogram showing the conversion rate of telomeric transgenes that are paternally inherited in the three different genetic contexts. *p*-values are indicated above corresponding bars (Pearson's Chi-squared test). n = ovariole number.

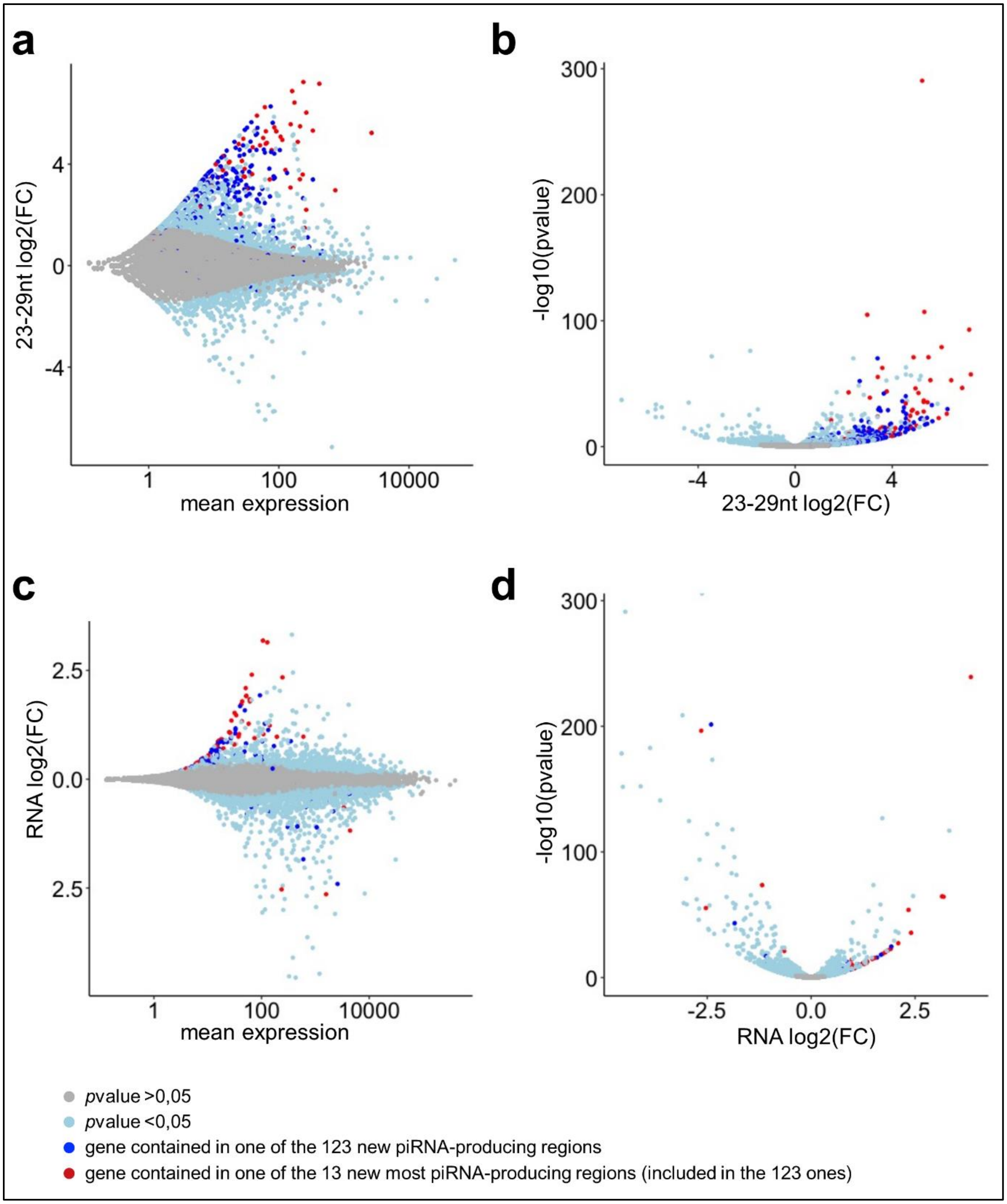

Figure S5. Global analyses of 23-29 nt and long RNA production in *Kdm3* GLKD compared to control ovaries. a, MA plot showing the differential genic production of small 23-29 nt RNA in *Kdm3* GLKD ovaries compared to control ovaries (from small RNA-seq data). Genes contained in the 13 most piRNA producing additional regions are in red, others genes of the 123 additional piRNA producing regions are in dark blue. Genes showing a differential production (p value < 0.05) are in light blue. Non significantly

differential producer genes are in grey. **b,** Volcano plot of the same data as in a. **c,** MA plot showing the differential expression of genes in *Kdm3* GLKD ovaries compared to control ovaries (from RNA-seq data). **d,** Volcano plot of the same data as in c.

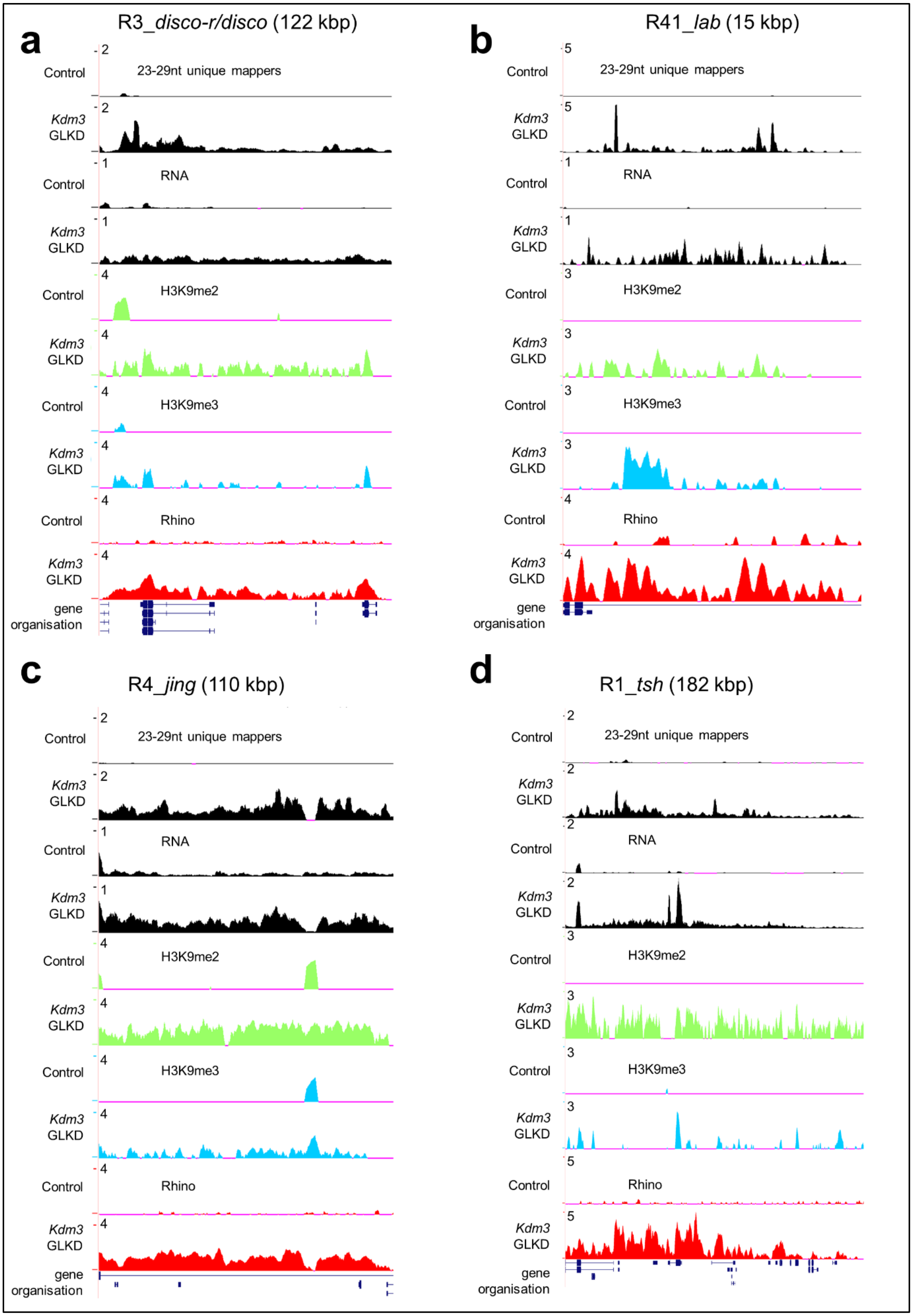
a
R3_disco-r/disco (122 kbp)
b
R41_lab (15 kbp)
c
R4_jing (110 kbp)
d
R1_tsh (182 kbp)
Control
Kdm3 GLKD
23-29nt unique mappers
RNA
H3K9me2
H3K9me3
Rhino
gene organisation

Figure S6. Panels summarizing *Kdm3* GLKD induced modifications of 23-29 nt production, RNAseq and ChIPseq analyses on several genomic regions including the *jing*, *lab*, *disco-r/disco* and *tsh* genes.

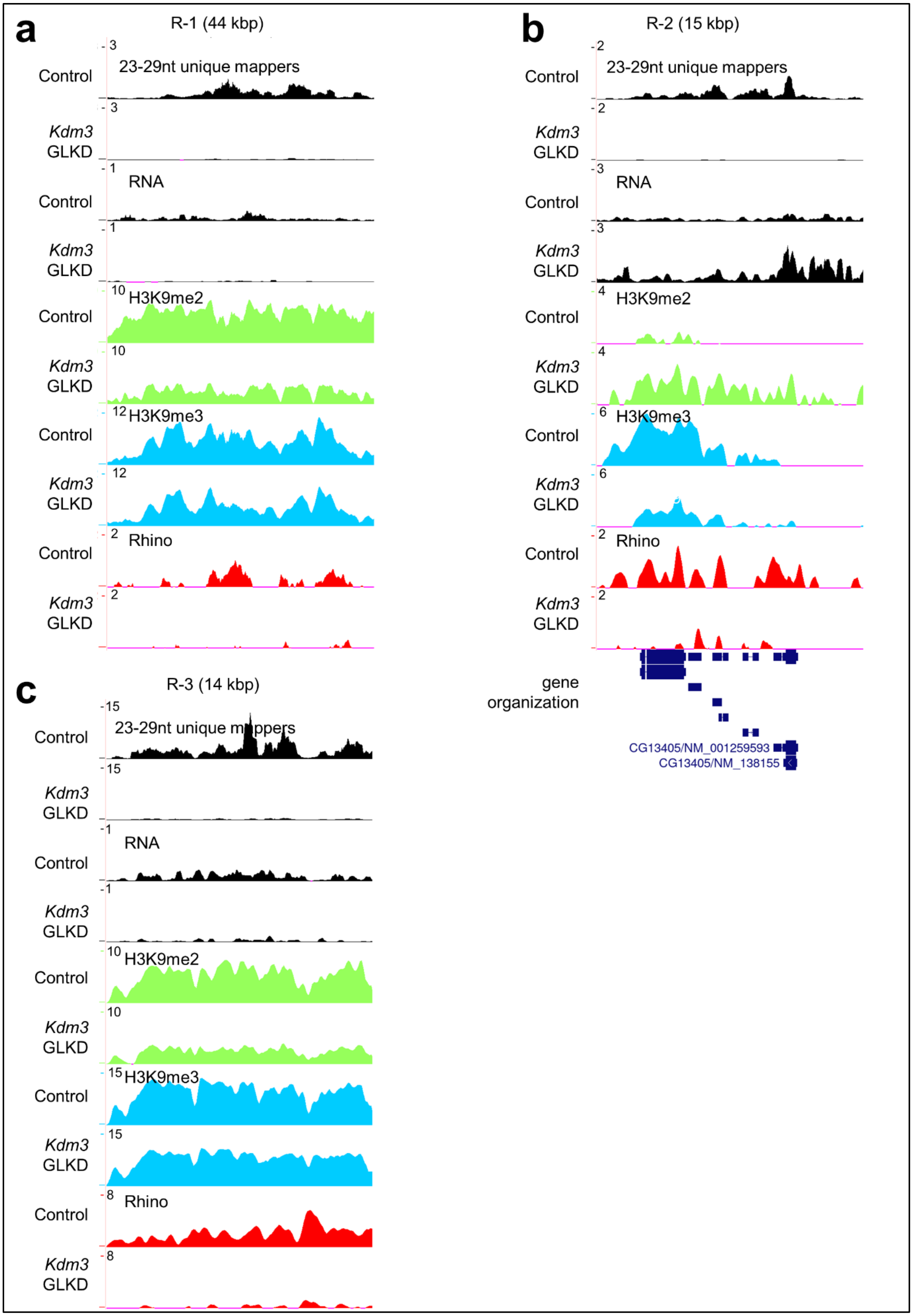
a
R-1 (44 kbp)
b
R-2 (15 kbp)
c
R-3 (14 kbp)
23-29nt unique mappers
RNA
H3K9me2
H3K9me3
Rhino
Control
Kdm3 GLKD
gene organization
CG13405/NM_001259593
CG13405/NM_138155

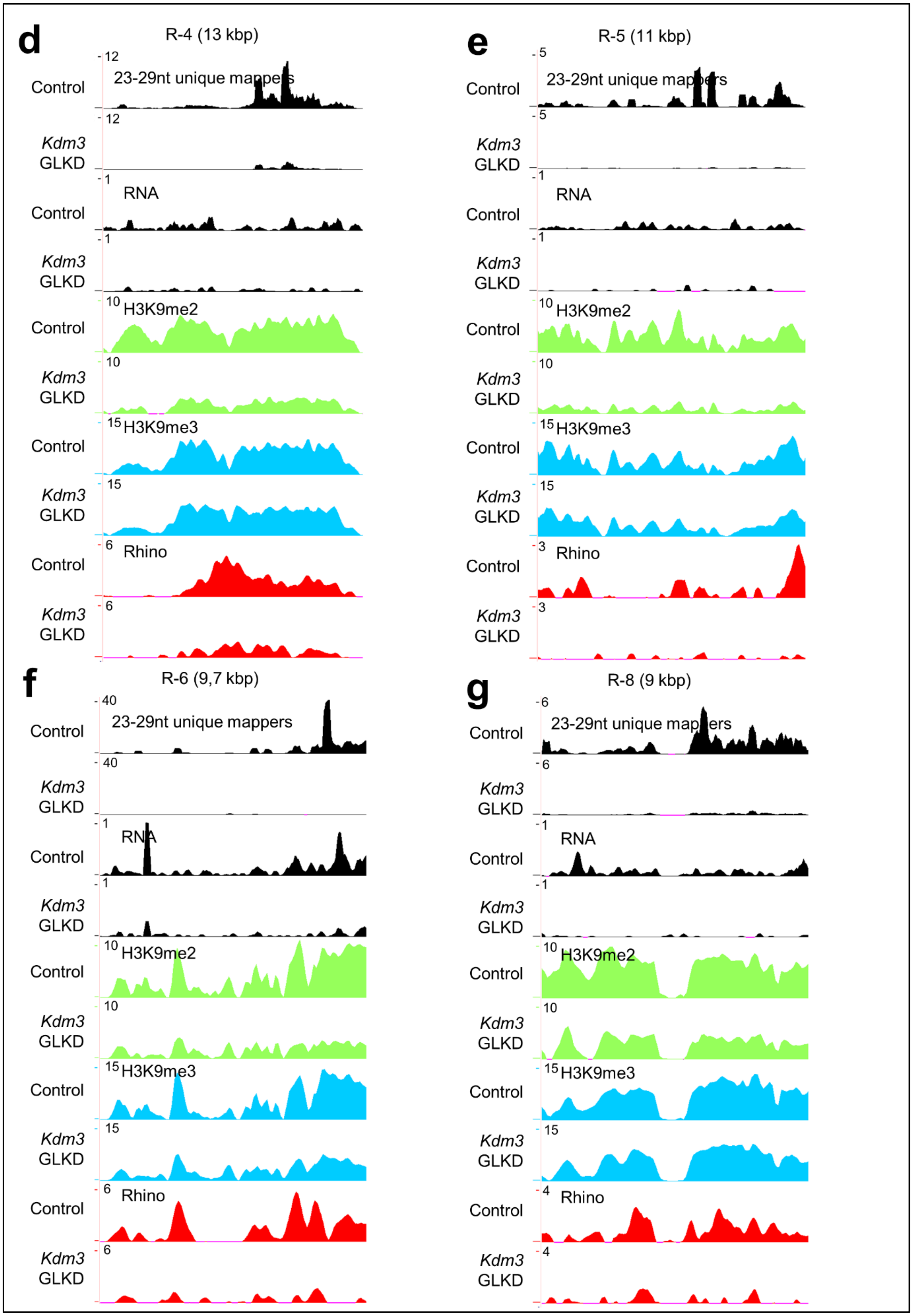
d
R-4 (13 kbp)
e
R-5 (11 kbp)
f
R-6 (9,7 kbp)
g
R-8 (9 kbp)
23-29nt unique mappers
RNA
H3K9me2
H3K9me3
Rhino
Control
Kdm3 GLKD

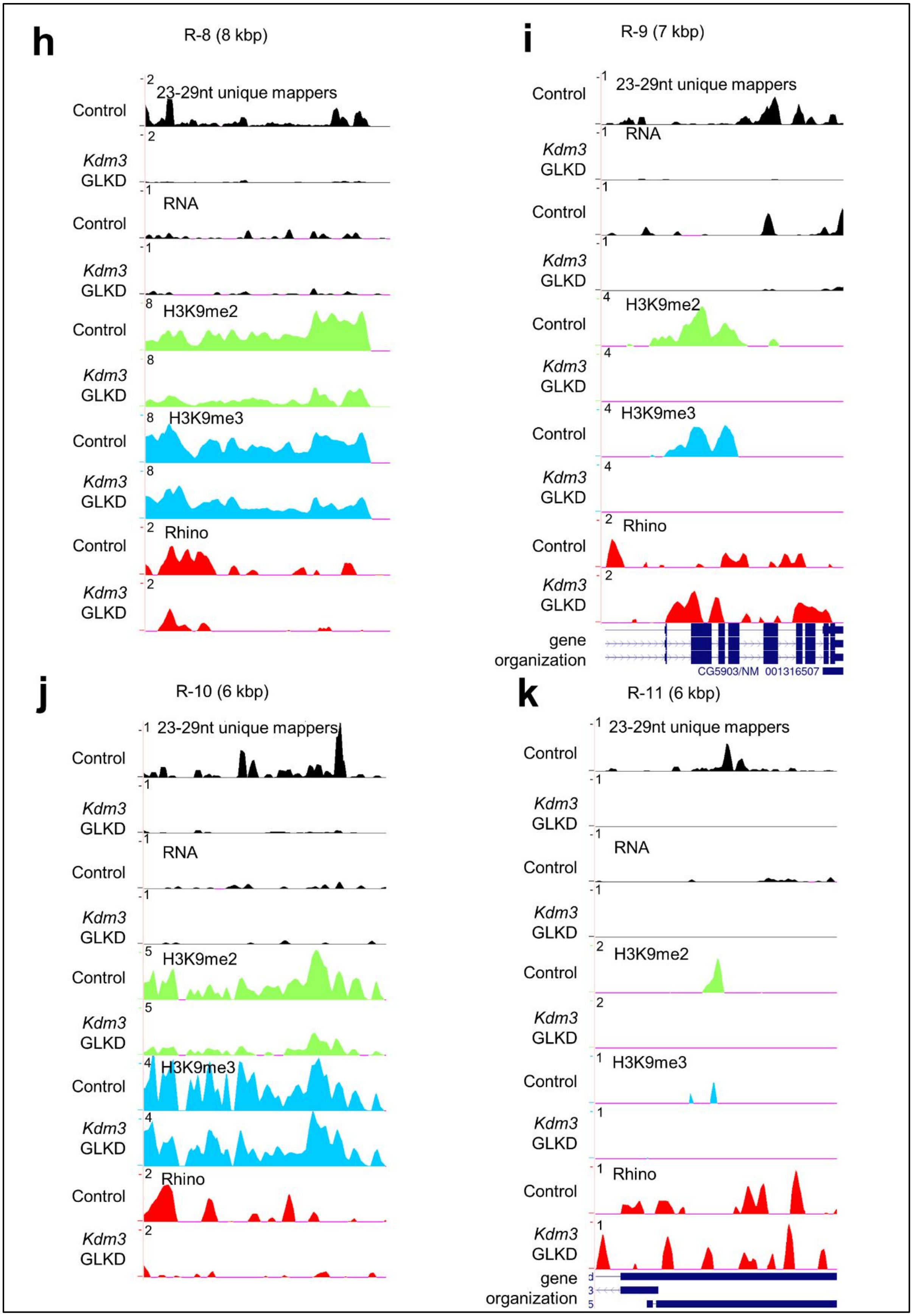

h
R-8 (8 kbp)
23-29nt unique mappers
Control
Kdm3 GLKD
RNA
H3K9me2
H3K9me3
Rhino
i
R-9 (7 kbp)
gene organization
CG5903/NM_001316507
j
R-10 (6 kbp)
k
R-11 (6 kbp)

Figure S7. Panels summarizing *Kdm3* GLKD induced modifications of 23-29 nt production, RNA-seq and ChIP-seq analyses on the eleven genomic regions that produce less piRNA in *Kdm3* GLKD. The number and the size of each region are indicated above each panel. When the gene organization is not shown, this is because there is no gene in the region.

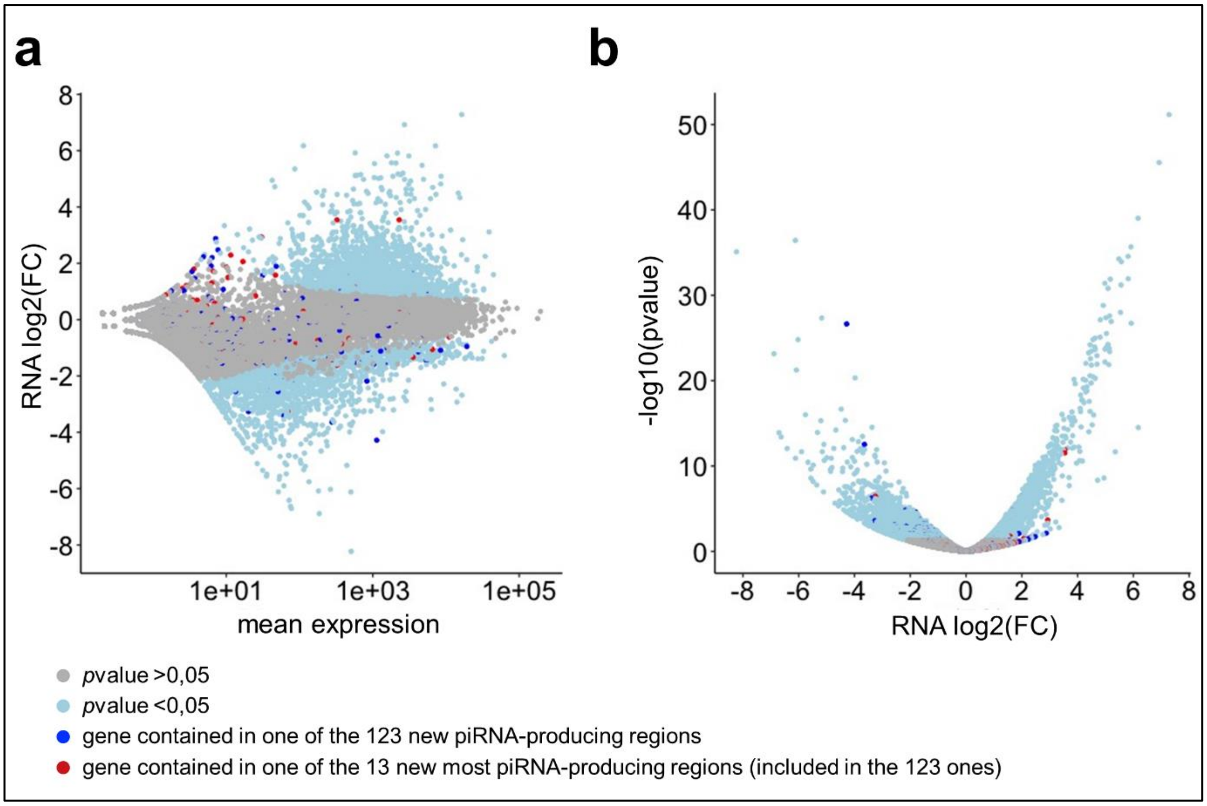

Figure S8. Global analyses of RNA production in *Kdm3* GLKD progeny compared to control (3-5 h embryos). a, MA plot showing the differential production of genic RNA in 3-5 h embryos coming from either *Kdm3* GLKD females or *w* GLKD females as control (from RNA-seq data). Genes contained in the 13 most piRNA producing additional regions are in red, others genes of the 123 additional piRNA producing regions are in dark blue. Genes showing a significant differential expression (p value < 0.05) are in light blue. Non significantly differential expressed genes are in grey. **b,** Volcano plot of the same data as in **a**.

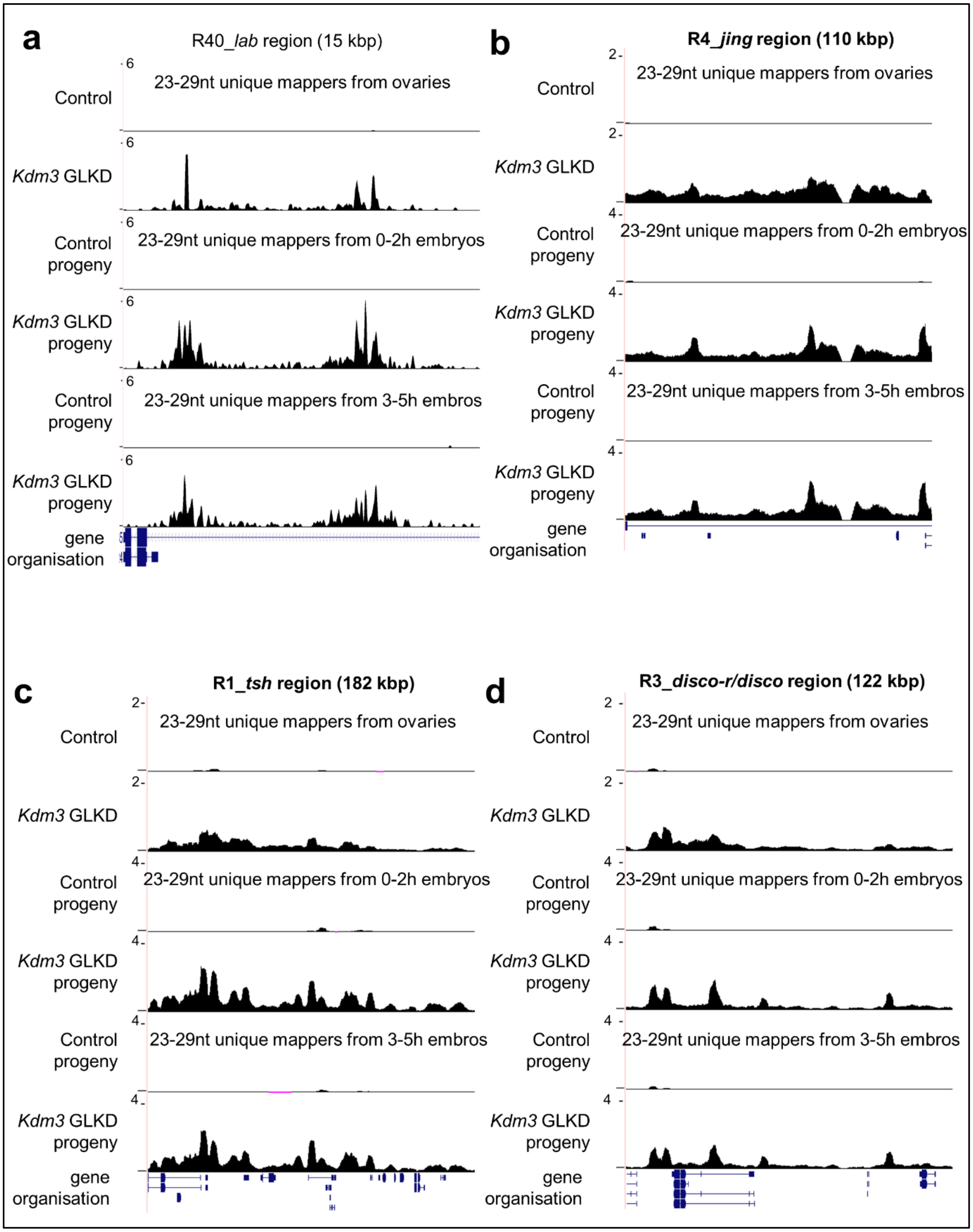

Figure S9. Summary maps of small RNA-seq (23-29 nucleotides) unique mappers from control or *Kdm3* GLKD ovaries or from their respective progeny, 0-2 h or 3-5 h embryos on genomic regions including *lab* (**a**), *jing* (**b**), *tsh* (**c**) *and disco-r/disco* (**d**) genes. Y-axis values are in rpm.

Table S1. List of tested shRNA lines. Gene name, gene symbol, CG number, Bloomington stock number, gonad phenotype and progeny viability are indicated.

Gene name	Gene symbol	CG	Bloomington stock	Gonad phenotype	Viable progeny
absent, small, or homeotic discs 1	ash1	CG8887	33705	Normal	yes
absent, small, or homeotic discs 1	ash1	CG8887	36803	Normal	yes
absent, small, or homeotic discs 2	ash2	CG6677	35388	Normal	no
Ada2a-containing complex component 2	Atac2	CG10414	32890	Normal	yes
Ada2a-containing complex component 2	Atac2	CG10414	53918	Atrophy	no
Additional sex combs	Asx	CG8787	51677	Normal	yes
adrift	aft	CG5032	63639	Normal	yes
alan shepard	shep	CG32423	33996	Normal	yes
alan shepard	shep	CG32423	38218	Normal	yes
alan shepard	shep	CG32423	43545	Normal	yes
Andropin	Anp	CG1361	55385	Normal	yes
antimeros	atms	CG2503	12114	Normal	yes
antisense RNA:CR45485	asRNA:CR45485	CR45485	62273	Normal	yes
archipelago	ago	CG15010	34802	Normal	yes
Argonaute 2	AGO2	CG7439	34799	Normal	yes
Argonaute 3	AGO3	CG40300	35232	Normal	no
Argonaute 3	AGO3	CG40300	44543	Normal	no
Argonaute-1	AGO1	CG6671	33727	Normal	no
ATP-dependent chromatin assembly factor large subunit	Acf1	CG1966	35575	Normal	yes
ATP-dependent chromatin assembly factor large subunit	Acf1	CG1966	35575	Normal	yes
Autophagy-related 1	Atg1	CG10967	44034	Normal	yes
Autophagy-related 17	Atg17	CG1347	36918	Normal	yes
Autophagy-related 18a	Atg18a	CG7986	34714	Normal	yes
Autophagy-related 2	Atg2	CG1241	35177	Normal	yes
Autophagy-related 8a	Atg8a	CG32672	34340	Normal	yes
B52	B52	CG10851	37519	Normal	yes
basket	bsk	CG5680	32977	Normal	yes
basket	bsk	CG5680	35594	Normal	yes
basket	bsk	CG5680	36643	Normal	yes
basket	bsk	CG5680	53310	Normal	yes
belle	bel	CG9748	35185	Atrophy	no
belle	bel	CG9748	35302	Normal	yes
blanks	blanks	CG10630	33667	Normal	nd
brahma	brm	CG5942	34520	Normal	yes
brahma	brm	CG5942	35210	Normal	yes
brahma	brm	CG5942	35211	Normal	yes
Brahma associated protein 60kD	Bap60	CG4303	32503	Normal	yes
Brahma associated protein 60kD	Bap60	CG4303	33954	Normal	yes
brivido-3	brv3	CG13762	33763	Normal	yes
brivido-3	brv3	CG13762	36774	Normal	yes

bruno1	bru1	CG31762	35394	Atrophy	no
bruno1	bru1	CG31762	38983	Atrophy	no
bruno1	bru1	CG31762	44483	Normal	no
Caper	Caper	CG11266	44431	Normal	yes
cap-n-collar	cnc	CG43286	32863	Normal	yes
cap-n-collar	cnc	CG43286	40854	Normal	yes
Carbonic anhydrase 2	CAH2	CG6906	41836	Normal	yes
Carbonic anhydrase 2	CAH2	CG6906	65081	Normal	yes
CG10418	CG10418	CG10418	44458	Atrophy	no
CG10445	CG10445	CG10445	43137	Normal	yes
CG11447	CG11447	CG11447	43207	Normal	yes
CG12054	CG12054	CG12054	50511	Normal	yes
CG12054	CG12054	CG12054	50910	Normal	yes
CG1239	CG1239	CG1239	42825	Normal	yes
CG1239	CG1239	CG1239	42912	Atrophy	no
CG1239	CG1239	CG1239	44023	Atrophy	no
CG1239	CG1239	CG1239	44506	Atrophy	no
CG1239	CG1239	CG1239	55400	Normal	yes
CG12493	CG12493	CG12493	42791	Normal	yes
CG13397	CG13397	CG13397	51808	Normal	yes
CG14131	CG14131	CG14131	63716	Normal	yes
CG1673	CG1673	CG1673	38363	Normal	yes
CG17544	CG17544	CG17544	64632	Normal	yes
CG17724	CG17724	CG17724	35676	Normal	yes
CG18537	CG18537	CG18537	53960	Normal	yes
CG2926	CG2926	CG2926	34941	Normal	nd
CG3036	CG3036	CG3036	43179	Normal	yes
CG31075	CG31075	CG31075	50654	Normal	yes
CG31075	CG31075	CG31075	62535	Normal	yes
CG31262	CG31262	CG31262	53970	Normal	yes
CG40006	CG40006	CG40006	34691	Normal	yes
CG40160	CG40160	CG40160	44268	Normal	yes
CG42542	CG42542	CG42542	35736	Normal	yes
CG42588	CG42588	CG42588	63698	Normal	yes
CG4267	CG4267	CG4267	32332	Normal	yes
CG4267	CG4267	CG4267	56900	Normal	yes
CG43373	CG43373	CG43373	64884	Atrophy	no
CG4404	CG4404	CG4404	62415	Normal	yes
CG4461	CG4461	CG4461	53298	Normal	yes
CG5001	CG5001	CG5001	32392	Normal	yes
CG5728	CG5728	CG5728	36592	Normal	yes
CG6283	CG6283	CG6283	51498	Normal	yes
CG6415	CG6415	CG6415	51867	Normal	yes
CG7255	CG7255	CG7255	58352	Normal	yes
CG7409	CG7409	CG7409	51433	Normal	yes

CG7878	CG7878	CG7878	35229	Normal	yes
CG8778	CG8778	CG8778	36793	Normal	yes
CG9684	CG9684	CG9684	36880	Normal	yes
CG9925	CG9925	CG9925	35811	Normal	yes
Chitinase 2	Cht2	CG2054	35717	Normal	no
Chitinase 2	Cht2	CG2054	60369	Normal	yes
Chromatin assembly factor 1, p55 subunit	Caf1-55	CG4236	34069	Atrophy	no
Chromosome associated protein D3	Cap-D3	CG31989	36615	Normal	yes
Cleavage and polyadenylation specific factor 6	Cpsf6	CG7185	34804	Atrophy	no
CMP-sialic acid synthase	Csas	CG32220	54843	Normal	yes
Complex I intermediate-associated protein, 30 kDa	CIA30	CG7598	55660	Normal	yes
corkscrew	csw	CG3954	33619	Normal	yes
corkscrew	csw	CG3954	35215	Normal	no
corkscrew	csw	CG3954	35638	Normal	no
corkscrew	csw	CG3954	60448	Normal	yes
crossover suppressor on 3 of Gowen	c(3)G	CG17604	62969	Normal	yes
C-terminal Binding Protein	CtBP	CG7583	32889	Normal	no
cup	cup	CG11181	35406	Normal	no
Cyclic-AMP response element binding protein A	CrebA	CG7450	42562	Normal	yes
Cyclin T	CycT	CG6292	32976	Atrophy	no
Cyclin T	CycT	CG6292	35168	Normal	yes
Cyclin-dependent kinase 4	Cdk4	CG5072	36060	Normal	yes
Cyclin-dependent kinase 4	Cdk4	CG5072	57031	Normal	yes
Cyclin-dependent kinase 9	Cdk9	CG5179	34982	Atrophy	no
Cyclin-dependent kinase 9	Cdk9	CG5179	35323	Small ovaries	yes
Cyclin-dependent kinase 9	Cdk9	CG5179	41932	Normal	yes
Cystathionine beta-synthase	Cbs	CG1753	36767	Normal	yes
Cystathionine beta-synthase	Cbs	CG1753	41877	Normal	yes
Cytochrome P450-4d8	Cyp4d8	CG4321	58019	Atrophy	no
D-2-hydroxyglutaric acid dehydrogenase	D2hgdh	CG3835	53355	Normal	yes
Darkener of apricot	Doa	CG42320	50903	Normal	no
Dead box protein 80	Dbp80	CG17023	34682	Normal	yes
deadhead	dhd	CG4193	41857	Normal	yes
Death-associated inhibitor of apoptosis 1 / thread	Diap1 / th	CG12284	33597	Normal	yes
decapentaplegic	dpp	CG9885	33618	Normal	yes
decapentaplegic	dpp	CG9885	33767	Normal	yes
decapentaplegic	dpp	CG9885	35214	Normal	yes
Desaturase 1	Desat1	CG5887	35591	Normal	yes
Desaturase 1	Desat1	CG5887	37512	Normal	yes
diaphanous	dia	CG1768	33424	Normal	no
diaphanous	dia	CG1768	35479	Atrophy	no
Dicer-2	Dcr2	CG6493	33656	Normal	yes
DISCO Interacting Protein 1	DIP1	CG17686	35226	Normal	yes
DISCO Interacting Protein 1	DIP1	CG17686	35333	Normal	yes

DISCO Interacting Protein 2	DIP2	CG7020	34918	Normal	yes
DISCO Interacting Protein 2	DIP2	CG7020	42598	Normal	yes
DnaJ-like-1	Dnaj-1	CG10578	32899	Normal	yes
DnaJ-like-1	Dnaj-1	CG10578	32978	Normal	yes
DnaJ-like-2	Droj2	CG8863	36089	Normal	yes
Dodeca-satellite-binding protein 1	Dp1	CG5170	32872	Normal	yes
domino	dom	CG9696	34827	Atrophy	no
domino	dom	CG9696	38385	Atrophy	no
domino	dom	CG9696	38941	Normal	yes
domino	dom	CG9696	40914	Atrophy	no
domino	dom	CG9696	41674	Atrophy	no
dorsal	dl	CG6667	32934	Normal	no
dorsal	dl	CG6667	36650	Normal	no
dorsal	dl	CG6667	38905	Normal	no
DP transcription factor	Dp	CG4654	33372	Normal	yes
Dpt-YFP repressor by overexpression	Dyro	CG6175	62516	Normal	yes
drosha	drosha	CG8730	33657	Normal	nd
drosha	drosha	CG8730	35233	Normal	yes
Dual oxidase	Duox	CG3131	32903	Normal	yes
Dual oxidase	Duox	CG3131	33975	Normal	yes
Dual oxidase	Duox	CG3131	38907	Normal	yes
Dual oxidase	Duox	CG3131	38916	Normal	yes
eIF4AIII	eIF4AIII	CG7483	32444	Atrophy	no
eIF4AIII	eIF4AIII	CG7483	32907	Atrophy	no
eIF4AIII	eIF4AIII	CG7483	38202	Atrophy	no
Ejaculatory bulb protein III	PebIII	CG11390	55933	Normal	yes
encore	enc	CG10847	42797	Normal	yes
Enhancer of bithorax	E(bx)	CG32346	33658	Normal	Subno
Enhancer of Polycomb	E(Pc)	CG7776	35271	Atrophy	no
Enhancer of zeste	E(z)	CG6502	33659	Normal	nd
Enhancer of zeste	E(z)	CG6502	36068	Normal	no
enoki mushroom	enok	CG11290	40917	Normal	yes
enoki mushroom	enok	CG11290	41664	Normal	yes
enoki mushroom	enok	CG11290	42941	Normal	yes
Ets at 97D	Ets97D	CG6338	35749	Normal	yes
Ets at 97D	Ets97D	CG6338	36635	Normal	yes
eukaryotic translation initiation factor 4E homologous protein	eIF4EHP	CG33100	36876	Normal	nd
eukaryotic translation initiation factor 4E homologous protein	eIF4EHP	CG33100	43990	Normal	yes
female sterile (1) Yb	fs(1)Yb	CG2706	35181	Normal	yes
female sterile (1) Yb	fs(1)Yb	CG2706	35301	Normal	yes
flower	fwe	CG6151	34157	Normal	yes
flower	fwe	CG6151	43157	Normal	yes
Fmr1	Fmr1	CG6203	35200	Normal	yes
Frost	Fst	CG9434	33376	Normal	yes

G9a	G9a	CG2995	34817	Normal	yes
Gcn5 acetyltransferase / Pcaf	Gcn5 / Pcaf	CG4107	33981	Normal	yes
Gcn5 acetyltransferase / Pcaf	Gcn5 / Pcaf	CG4107	35601	Normal	no
Glutathione S transferase E1	GstE1	CG5164	36878	Normal	yes
Glutathione S transferase E1	GstE1	CG5164	36878	Normal	yes
Glutathione S transferase E6	GstE6	CG17530	65197	Normal	yes
half pint	hfp	CG12085	34785	Normal	yes
hangover	hang	CG32575	35674	Normal	yes
hangover	hang	CG32575	41870	Normal	no
haywire	hay	CG8019	53345	Atrophy	no
Heat shock 70-kDa protein cognate 3	Hsc70-3	CG4147	32402	Normal	yes
Heat shock factor	Hsf	CG5748	41581	Normal	yes
Heat shock gene 67Ba	Hsp67Ba	CG4167	41962	Normal	yes
Heat shock gene 67Ba	Hsp67Ba	CG4167	53007	Normal	yes
Heat shock gene 67Bc	Hsp67Bc	CG4190	35452	Normal	yes
Heat shock gene 67Bc	Hsp67Bc	CG4190	42607	Normal	yes
Heat shock protein 22	Hsp22	CG4460	41709	Normal	yes
Heat shock protein 22	Hsp22	CG4460	51397	Normal	yes
Heat shock protein 23	Hsp23	CG4463	44029	Normal	yes
Heat shock protein 26	Hsp26	CG4183	35408	Normal	yes
Heat shock protein 26	Hsp26	CG4183	42610	Normal	yes
Heat shock protein 27	Hsp27	CG4466	33007	Normal	yes
Heat shock protein 27	Hsp27	CG4466	33922	Normal	yes
Heat shock protein 60A	Hsp60A	CG12101	34729	Normal	yes
Heat shock protein 60B	Hsp60B	CG2830	34729	Normal	yes
Heat shock protein 67Bb	Hsp67Bb	CG4456	41709	Normal	yes
Heat shock protein 67Bb	Hsp67Bb	CG4456	51397	Normal	yes
Heat shock protein 68	Hsp68	CG5436	50637	Normal	yes
Heat shock protein 70Aa	Hsp70Aa	CG31366	42639	Normal	yes
Heat shock protein 70Ab	Hsp70Ab	CG18743	35663	Normal	yes
Heat shock protein 70Ba	Hsp70Ba	CG31449	32997	Normal	yes
Heat shock protein 70Ba	Hsp70Ba	CG31449	43289	Normal	yes
Heat shock protein 70Ba	Hsp70Ba	CG31449	35672	Normal	yes
Heat shock protein 70Bb	Hsp70Bb	CG31359	32997	Normal	yes
Heat shock protein 70Bb	Hsp70Bb	CG31359	33948	Normal	yes
Heat shock protein 70Bc	Hsp70Bc	CG6489	32997	Normal	yes
Heat shock protein 70Bc	Hsp70Bc	CG6489	35697	Normal	yes
Heat shock protein 70Bc	Hsp70Bc	CG6489	42626	Normal	yes
Heat shock protein 83	Hsp83	CG1242	32996	Normal	no
Heat shock protein 83	Hsp83	CG1242	33947	Atrophy	no
Heat shock protein cognate 1	Hsc70-1	CG8937	34527	Normal	yes
Heat shock protein cognate 2	Hsc70-2	CG7756	42014	Normal	yes
Heat shock protein cognate 2	Hsc70-2	CG7756	44485	Normal	yes
Heat shock protein cognate 2	Hsc70-2	CG7756	32997	Normal	yes
Heat shock protein cognate 4	Hsc70-4	CG4264	34836	Atrophy	no

Heat shock protein cognate 4	Hsc70-4	CG4264	35684	Atrophy	no
Helicase at 25E	Hel25E	CG7269	33666	Atrophy	no
hemipterous	hep	CG4353	35210	Normal	no
hephaestus	heph	CG31000	35669	Normal	no
hephaestus	heph	CG31000	55655	Normal	yes
Heterochromatin Protein 1b	HP1b	CG7041	32401	Normal	yes
Heterogeneous nuclear ribonucleoprotein at 27C	Hrb27C	CG10377	33716	Normal	no
Heterogeneous nuclear ribonucleoprotein at 87F	Hrb87F	CG12749	52937	Normal	yes
Heterogeneous nuclear ribonucleoprotein at 98DE	Hrb98DE	CG9983	32351	Atrophy	no
Heterogeneous nuclear ribonucleoprotein K/bancal	HnRNP-K/bl	CG13425	42540	Normal	yes
Host cell factor	Hcf	CG1710	32453	Normal	yes
Host cell factor	Hcf	CG1710	36799	Normal	yes
Hsp70Bbb	Hsp70Bbb	CG5834	32997	Normal	yes
Hsp70Bbb	Hsp70Bbb	CG5834	33000	Normal	yes
Hsp70Bbb	Hsp70Bbb	CG5834	33916	Atrophy	no
HSPB1 associated protein 1	HSPBAP1	CG43320	34606	Normal	yes
Imaginal disc growth factor 2	Idgf2	CG4475	55935	Normal	yes
Imitation SWI	Iswi	CG8625	32845	Normal	nd
Imitation SWI	Iswi	CG8625	51931	Normal	yes
Insulin-like peptide 5	Ilp5	CG33273	33683	Normal	yes
Inwardly rectifying potassium channel 1	Irk1	CG44159	42644	Normal	yes
Isocitrate dehydrogenase	Idh	CG7176	41708	Normal	yes
Jabba	Jabba	CG42351	36852	Normal	no
janus A	janA	CG7933	41846	Normal	yes
JIL-1 anchoring and stabilizing protein	Jasper	CG7946	55274	Normal	nd
JIL-1 kinase	JIL-1	CG6297	41592	Normal	yes
JIL-1 kinase	JIL-1	CG6297	42571	ND	nd
JIL-1 kinase	JIL-1	CG6297	55875	Normal	yes
JIL-1 kinase	JIL-1	CG6297	57293	Normal	nd
jing interacting gene regulatory 1	jigr1	CG17383	58173	Normal	yes
Jumonji domain containing 5	JMJD5	CG13902	33702	Normal	yes
Jumonji, AT rich interactive domain 2	jarid2	CG3654	32891	Normal	yes
Juvenile hormone epoxide hydrolase 3	Jheh3	CG15106	60021	Normal	no
Kank	Kank	CG10249	33432	Normal	yes
kayak	kay	CG33956	33379	Normal	no
Keap1	Keap1	CG3962	40932	Normal	no
kismet	kis	CG3696	34908	Normal	yes
kismet	kis	CG3696	36597	Normal	no
kismet	kis	CG3696	44542	Normal	no
kohtalo	kto	CG8491	34588	Normal	no
krimper	krimp	CG15707	35230	Normal	no
krimper	krimp	CG15707	35231	Normal	no
krimper	krimp	CG15707	37511	Normal	no
Lamin	Lam	CG6944	36617	Normal	yes
lethal (2) essential for life	l(2)efl	CG4533	41724	Normal	yes

lethal (2) essential for life	l(2)efl	CG4533	51816	Normal	yes
lethal (3) 72Ab	l(3)72Ab	CG5931	34024	Atrophy	no
lethal (3) 72Ab	l(3)72Ab	CG5931	50716	Atrophy	no
lethal (3) 80Fg	l(3)80Fg	CG40178	44578	Normal	yes
Leucine zipper and EF-hand containing transmembrane protein 1	Letm1	CG4589	37502	Atrophy	no
Ligase4	lig4	CG12176	51933	Normal	yes
little imaginal discs	lid	CG9088	36652	Normal	yes
little imaginal discs	lid	CG9088	35706	Normal	yes
locomotion defects	loco	CG5248	32456	Normal	yes
loki	lok	CG10895	35152	Normal	no
longitudinals lacking	lola	CG12052	35721	Normal	yes
loquacious	loqs	CG6866	32955	Normal	yes
loquacious	loqs	CG6866	33427	Normal	yes
loquacious	loqs	CG6866	34779	Normal	yes
loquacious	loqs	CG6866	34780	Normal	yes
loquacious	loqs	CG6866	34781	Normal	yes
loquacious	loqs	CG6866	34782	Normal	yes
loquacious	loqs	CG6866	34851	Normal	yes
Lysine (K)-specific demethylase 2	Kdm2	CG11033	33699	Normal	yes
Lysine (K)-specific demethylase 3	Kdm3	CG8165	32975	Normal	no
Lysine (K)-specific demethylase 4A	Kdm4A	CG15835	34629	Normal	yes
Lysine (K)-specific demethylase 4B	Kdm4B	CG33182	35676	Normal	yes
mago nashi	mago	CG9401	35453	Normal	yes
Major Facilitator Superfamily Transporter 17	MFS17	CG40263	44033	Normal	yes
maleless	mle	CG11680	34864	Normal	nd
maternal expression at 31B	Me31b	CG4916	33675	Atrophy	no
maternal expression at 31B	Me31b	CG4916	38923	Normal	yes
Max	Max	CG9648	40851	Normal	yes
meiotic 41	mei-41	CG4252	35371	Normal	yes
meiotic 41	mei-41	CG4252	41934	Normal	yes
meiotic 9	mei-9	CG3697	55313	Normal	yes
Mekk1	Mekk1	CG7717	35402	Normal	yes
menage a trois	metro	CG30021	35810	Normal	yes
Menin 1	Mnn1	CG13778	35150	Atrophy	no
Menin 1	Mnn1	CG13778	51862	Normal	yes
methuselah	mth	CG6936	36823	Normal	yes
Methyltransferase 2	Mt2	CG10692	38224	Normal	yes
Methyltransferase 2	Mt2	CG10692	42906	Normal	yes
Mi-2	Mi-2	CG8103	35398	Atrophy	no
Mi-2	Mi-2	CG8103	33419	Atrophy	no
Mi-2	Mi-2	CG8103	51774	Atrophy	no
moira	mor	CG18740	34919	Normal	yes
moira	mor	CG18740	35630	Normal	yes
moira	mor	CG18740	35662	Normal	no

Mucin related 18B	Mur18B	CG7874	56957	Normal	yes
mushroom-body expressed	mub	CG7437	34870	Normal	yes
nejire	nej	CG15319	36682	Normal	yes
nejire	nej	CG15319	37489	Normal	no
Neurofibromin 1	Nf1	CG8318	53322	Normal	yes
Nucleolar protein 66	NO66	CG2982	33596	Normal	yes
Nucleosome remodeling factor - 38kD	Nurf-38	CG4634	35444	Normal	no
osa	osa	CG7467	35447	Normal	yes
osa	osa	CG7467	38285	Normal	yes
ovarian tumor	otu	CG12743	34065	Atrophy	no
ovaries absent	ova	CG5694	36655	Normal	yes
ovaries absent	ova	CG5694	62485	Normal	yes
p23	p23	CG16817	41862	Normal	yes
p24-related-2	p24-2	CG33105	40839	Normal	yes
p38a MAP kinase	p38a	CG5475	34744	Normal	yes
p38a MAP kinase	p38a	CG5475	35244	Normal	yes
p38b MAP kinase	p38b	CG7393	35252	Normal	yes
p53	p53	CG10895	36814	Normal	yes
p53	p53	CG10895	41638	Normal	yes
p53	p53	CG10895	41720	Normal	yes
pacman	pcm	CG3291	34690	Normal	yes
painless	pain	CG15860	51835	Normal	yes
pancreatic eIF-2α kinase	PEK	CG2087	35162	Normal	yes
pancreatic eIF-2α kinase	PEK	CG2087	42499	Normal	yes
papi	papi	CG7082	34932	Normal	yes
papi	papi	CG7082	35450	Normal	yes
papi	papi	CG7082	37513	Normal	yes
papi	papi	CG7082	38216	Normal	yes
partner of drosha	pasha	CG1800	33972	Normal	nd
peanuts	pea	CG8241	32838	Atrophy	no
P-element induced wimpy testis	piwi	CG6122	33724	Atrophy	no
P-element induced wimpy testis	piwi	CG6122	34866	Atrophy	no
P-element induced wimpy testis	piwi	CG6122	37483	Normal	no
P-element somatic inhibitor	Psi	CG8912	34825	Normal	yes
Phosphoglycerate mutase 5	Pgam5	CG14816	33346	Normal	yes
Phosphotidylinositol 3 kinase 59F	Pi3K59F	CG5373	33384	Normal	yes
Phosphotidylinositol 3 kinase 59F	Pi3K59F	CG5373	36056	Normal	yes
Poly-(ADP-ribose) polymerase	Parp	CG40411	35792	Normal	yes
Polycomb	Pc	CG32443	33622	Normal	yes
Polycomb	Pc	CG32443	33964	Normal	yes
Polycomb	Pc	CG32443	36070	Normal	yes
Polycomblike	Pcl	CG5109	33945	Normal	yes
Polycomblike	Pcl	CG5109	33946	Normal	yes
polyhomeotic proximal	ph-p	CG18412	33669	Normal	yes
pontin	pont	CG4003	50972	Atrophy	no

pre-mRNA processing factor 40	Prp40	CG3542	33711	Normal	yes
pre-mRNA processing factor 6	Prp6	CG6841	51909	Atrophy	no
Prip	Prip	CG7777	44464	Normal	yes
Prip	Prip	CG7777	50695	Normal	yes
protein partner of snf	pps	CG6525	38529	Normal	yes
protein partner of snf	pps	CG6525	38912	Normal	yes
puckered	puc	CG7850	53019	Normal	yes
puckered	puc	CG7850	34392	Normal	yes
puckered	puc	CG7850	36085	Normal	no
puckered	puc	CG7850	44038	Normal	yes
punt	put	CG7904	35195	Normal	no
punt	put	CG7904	35701	Normal	yes
punt	put	CG7904	39025	Normal	yes
pyrexia	pyx	CG17142	51836	Normal	yes
qin	qin	CG43726	37475	Normal	yes
qin	qin	CG43726	41662	Normal	nd
r2d2	r2d2	CG7138	34784	Normal	yes
Rab5	Rab5	CG3664	34832	Normal	no
Rab5	Rab5	CG3664	51847	Normal	yes
raspberry	ras	CG1799	51717	Small ovaries	yes
Ras-related protein interacting with calmodulin	Ric	CG8418	41819	Normal	yes
Recombination repair protein 1	Rrp1	CG3178	35420	Normal	yes
refractory to sigma P	ref(2)P	CG10360	33978	Normal	yes
refractory to sigma P	ref(2)P	CG10360	36111	Normal	yes
Regulator of telomere elongation helicase 1	Rtel1	CG4078	32973	Normal	yes
Rev1	Rev1	CG12189	36654	Normal	yes
rhino	rhi	CG10683	34071	Normal	yes
rhino	rhi	CG10683	35171	Normal	no
RhoGAP71E	RhoGAP71E	CG32149	32417	Normal	yes
rho-type guanine exchange factor	rtGEF	CG10043	32947	Normal	yes
Ribosomal protein L8	Rpl8	CG1263	50610	Normal	yes
Rm62	Rm62	CG10279	34829	Normal	yes
RNA polymerase II subunit Rpb4	Rpb4	CG43662	50905	Atrophy	no
rotated abdomen	rt	CG6097	51805	Normal	yes
Rox8	Rox8	CG5422	32472	Normal	yes
Rpd3	Rpd3	CG7471	33725	Normal	yes
Rpd3	Rpd3	CG7471	34846	Normal	yes
Rrp6	Rrp6	CG7292	34809	Letal	nd
Rrp6	Rrp6	CG7292	42064	Normal	no
runt	run	CG1849	34707	Normal	yes
sans fille	snf	CG4528	34593	Normal	yes
sans fille	snf	CG4528	51459	Atrophy	no
Scaffold attachment factor B	Saf-B	CG6995	51759	Normal	no
Scamp	Scamp	CG9195	38277	Normal	yes

scute	sc	CG3827	41594	Normal	yes
Secreted protein, acidic, cysteine-rich	SPARC	CG6378	40885	Normal	yes
Serine palmitoyltransferase subunit I	Spt-I	CG4016	55685	Normal	yes
SET domain binding factor	Sbf	CG6939	32419	Normal	yes
SET domain binding factor	Sbf	CG6939	44004	Normal	yes
Sex lethal	Sxl	CG43770	34393	Atrophy	no
Sex lethal	Sxl	CG43770	38195	Atrophy	no
shibire	shi	CG18102	36921	Normal	no
shotgun	shg	CG3722	32904	Normal	yes
shotgun	shg	CG3722	38207	Normal	no
shriveled	shv	CG4164	37507	Normal	yes
shriveled	shv	CG4164	54797	Normal	yes
shutdown	shu	CG4735	35454	Normal	no
Sin3A	Sin3A	CG8815	32368	Normal	yes
sisterless A	sisA	CG1641	55181	Normal	yes
skuld	skd	CG9936	34630	Normal	yes
slipper	slpr	CG2272	32948	Normal	yes
slipper	slpr	CG2272	41605	Normal	yes
small ribonucleoprotein particle U1 subunit 70K	snRNP-U1-70K	CG8749	33396	Atrophy	no
small ribonucleoprotein particle U1 subunit C	snRNP-U1-C	CG5454	34822	Atrophy	no
smaug	smg	CG5263	35477	Normal	yes
Snf5-related 1	Snr1	CG1064	32372	Normal	no
Son RNA binding protein	Son	CG8273	34805	Normal	yes
Sorbitol dehydrogenase-2	Sodh-2	CG4649	53353	Normal	Few yes
spaghetti squash	sqh	CG3595	32439	ND	nd
spaghetti squash	sqh	CG3595	33892	Normal	yes
spaghetti squash	sqh	CG3595	38222	Normal	yes
Spf45	Spf45	CG17540	41954	Normal	yes
spindle A	Spn-A	CG7948	38898	Normal	yes
spindle A	Spn-A	CG7948	51936	Normal	yes
spindle E	spn-E	CG3158	35303	Normal	no
Splicing factor 2	SF2	CG6987	32367	Normal	yes
Splicing factor 30	Spf30	CG17454	43199	Atrophy	no
Splicing factor 3a subunit 1	Sf3a1	CG16941	34840	Atrophy	no
Spt3	Spt3	CG3169	35148	Normal	yes
Spt4	Spt4	CG12372	32896	Atrophy	no
Spt5	Spt5	CG7626	34837	Atrophy	no
Spt6	Spt6	CG12225	32373	Atrophy	no
Spt7	Spt7	CG6506	42552	Normal	yes
squid	sqd	CG16901	35627	Normal	yes
squid	sqd	CG16901	53891	Normal	yes
staufen	stau	CG5753	35690	Normal	yes
staufen	stau	CG5753	43187	Normal	no
stonewall	stwl	CG3836	35415	Atrophy	no

Su(var)2-HP2	Su(var)2-HP2	CG12864	38255	Atrophy	no
suppressor of Hairy wing	su(Hw)	CG8573	33906	Normal	yes
suppressor of Hairy wing	su(Hw)	CG8573	34006	Normal	yes
Suppressor of sable	su(sable)	CG6222	33982	Normal	yes
Suppressor of Under-Replication	SuUR	CG7869	36893	Normal	yes
Suppressor of variegation 205	Su(var)205	CG8409	33400	Atrophy	no
Suppressor of variegation 205	Su(var)205	CG8409	36792	Atrophy	no
Suppressor of variegation 2-10	Su(var)2-10	CG8068	32915	Atrophy	no
Suppressor of variegation 2-10	Su(var)2-10	CG8068	32956	Atrophy	no
Suppressor of variegation 2-10	Su(var)2-10	CG8068	58067	Atrophy	no
Suppressor of variegation 3-3	Su(var)3-3	CG17149	32853	Normal	yes
Suppressor of variegation 3-3	Su(var)3-3	CG17149	33726	Normal	yes
Suppressor of variegation 3-3	Su(var)3-3	CG17149	36867	Normal	yes
Suppressor of variegation 3-9	Su(var)3-9	CG43664	32914	Atrophy	no
Suppressor of variegation 3-9	Su(var)3-9	CG43664	33401	Atrophy	no
Suppressor of variegation 3-9	Su(var)3-9	CG43664	43661	Atrophy	no
Suppressor of zeste 2	Su(z)2	CG3905	33403	Normal	yes
survival motor neuron	Smn	CG16725	36621	Normal	yes
tapas	tapas	CG8920	34738	Normal	yes
TBP-associated factor 1	Taf1	CG17603	32421	Atrophy	no
TBP-associated factor 1	Taf1	CG17603	35314	Atrophy	no
TBP-associated factor 10	Taf10	CG2859	35239	Normal	yes
TBP-associated factor 12	Taf12	CG17358	34852	Normal	yes
TBP-associated factor 4	Taf4	CG5444	35427	Normal	no
TBP-associated factor 4	Taf4	CG5444	50985	Atrophy	no
TBP-associated factor 5	Taf5	CG7704	35367	Atrophy	no
TBP-associated factor 7	Taf7	CG2670	55216	Normal	yes
tejas	tej	CG8589	36879	Normal	no
tejas	tej	CG8589	41929	Normal	no
thickveins	tkv	CG14026	35166	Normal	yes
thickveins	tkv	CG14026	35653	Normal	yes
thickveins	tkv	CG14026	40937	Atrophy	no
thickveins	tkv	CG14026	41904	Normal	yes
Thioredoxin peroxidase 2	Jafrac2	CG1274	56043	Normal	yes
Thor	Thor	CG8846	36667	Normal	yes
Thor	Thor	CG8846	36815	Normal	yes
transcriptional Adaptor 2a	Ada2a	CG43663	50905	Atrophy	no
transcriptional Adaptor 2b	Ada2b	CG9638	35334	Normal	yes
transcriptional Adaptor 3	Ada3	CG7098	32451	Normal	yes
Transient receptor potential cation channel A1	TrpA1	CG5751	36780	Normal	yes
trithorax	trx	CG8651	33703	Normal	yes
tsunagi	tsu	CG8781	36585	Atrophy	no
tudor	tud	CG9450	42800	Normal	yes
Tudor domain containing 3	Tdrd3	CG13472	36819	Normal	yes
Tudor staphylococcal nuclease	Tudor-SN	CG7008	34865	Normal	nd

Turandot A	TotA	CG31509	53244	Normal	yes
Turandot A	TotA	CG31509	55378	Normal	yes
Turandot A	TotA	CG31509	58357	Normal	yes
Turandot C	TotC	CG31508	51407	Normal	yes
U2 small nuclear riboprotein auxiliary factor 38	U2af38	CG3582	50561	Atrophy	no
U2 small nuclear riboprotein auxiliary factor 50	U2af50	CG9998	50521	Normal	no
Uncoordinated 115a	Unc-115a	CG31352	40839	Normal	yes
Uncoordinated 115b	Unc-115b	CG31332	40839	Normal	yes
Upf1	Upf1	CG1559	43144	Normal	no
Upf3	Upf3	CG11184	44565	Normal	nd
upSET	upSET	CG9007	51447	Normal	yes
Utx histone demethylase	Utx	CG5640	34076	Normal	yes
vasa	vas	CG46283	32434	Normal	no
vasa intronic gene	vig	CG4170	35183	Normal	no
vasa intronic gene	vig	CG4170	35184	Normal	yes
Victoria	Victoria	CG33117	55953	Normal	yes
walrus	wal	CG8996	34915	Normal	yes
windei	wde	CG12340	33339	Normal	nd
XNP	XNP	CG4548	32894	Normal	yes
β Hydroxy acid dehydrogenase 1	Had1	CG9914	62273	Normal	yes
β-Mannosidase	β-Man	CG12582	53272	Normal	yes

Table S2. Summary of small RNA-seq, RNA-seq and ChIP-seq data. Name of library, genotype and depth are given. The fastq.gz files were deposited on the GEO under the number GSE203279.

Library ID	Purpose	Genotype	Tissue	D. mel dm6 multimappers (depth)	Normalization factor (rpm)	D. mel dm6 clean 23-29 nt unique mappers
GRH-103	small seq	hfp GLKD	ovary	8630134	0.116	
GRH-104	small seq	progeny of hfp GLKD	ovary	12107109	0.083	
GRH-105	small seq	hfp control sisters	ovary	10057434	0.099	
GRH-106	small seq	progeny of hfp control sisters	ovary	16237313	0.062	
GRH-111	small seq	Kdm3 GLKD	ovary	11418729	0.088	
GRH-112	small seq	Kdm3 control sisters	ovary	14726077	0.068	
ALBA-28	small seq	Kdm3 GLKD	ovary	20771697	0.048	1287136
ALBA-29	small seq	Kdm3 GLKD	ovary	29582387	0.034	1996135
ALBA-30	small seq	Kdm3 GLKD	ovary	25565165	0.039	637207
ALBA-25	small seq	Kdm3 control sisters	ovary	24013259	0.042	1479172
ALBA-26	small seq	Kdm3 control sisters	ovary	29159111	0.034	1754599
ALBA-27	small seq	Kdm3 control sisters	ovary	23356824	0.043	1400470
ALBA-39	small seq	w GLKD progeny	0-2h embryos	4010995	0,249	336889
ALBA-40	small seq	w GLKD progeny	0-2h embryos	7539003	0,132	658483
ALBA-41	small seq	Kdm3 GLKD progeny	0-2h embryos	5977845	0,167	693285
ALBA-42	small seq	Kdm3 GLKD progeny	0-2h embryos	11020010	0,091	1300373
ALBA-43	small seq	w GLKD progeny	3-5h embryos	6941014	0,144	344605
ALBA-44	small seq	w GLKD progeny	3-5h embryos	5234332	0,191	306237
ALBA-45	small seq	Kdm3 GLKD progeny	3-5h embryos	9206467	0,108	876956
ALBA-46	small seq	Kdm3 GLKD progeny	3-5h embryos	5290196	0,189	577108
Library ID	Purpose	Genotype	Tissue	D. mel. dm6 reads	D. mel R6.36 all-genes reads	
ALBA-1	RNA seq	Kdm3 GLKD	ovary	29044815	25268570	
ALBA-2	RNA seq	Kdm3 GLKD	ovary	41170212	34401515	
ALBA-3	RNA seq	Kdm3 GLKD	ovary	26531156	22946996	
ALBA-4	RNA seq	Kdm3 control sisters	ovary	33133242	27776026	
ALBA-5	RNA seq	Kdm3 control sisters	ovary	43201907	37041176	
ALBA-6	RNA seq	Kdm3 control sisters	ovary	38095053	31937164	
ALBA-51	RNA seq	w GLKD progeny	3-5h embryos	23069851	18999084	
ALBA-52	RNA seq	w GLKD progeny	3-5h embryos	17326162	14704621	
ALBA-53	RNA seq	Kdm3 GLKD progeny	3-5h embryos	26322424	23468478	
ALBA-54	RNA seq	Kdm3 GLKD progeny	3-5h embryos	21808124	20230925	
Library ID	Purpose	Genotype	Tissue	Antibody	D. mel. dm6 reads	D. mel. dm6 clean
ADQN-78	ChIP seq	Kdm3 control sisters	ovary	no (INPUT for me2 and me3)	38214851	37622419
ADQN-79	ChIP seq	Kdm3 GLKD	ovary	no (INPUT for me2 and me3)	31085861	30561343
ADQN-80	ChIP seq	Kdm3 control sisters	ovary	αH3K9me2	17429811	15880104
ADQN-81	ChIP seq	Kdm3 control sisters	ovary	αH3K9me2	19637657	18181965
ADQN-82	ChIP seq	Kdm3 control sisters	ovary	αH3K9me2	21020633	19405338
ADQN-83	ChIP seq	Kdm3 GLKD	ovary	αH3K9me2	19386591	18602355
ADQN-84	ChIP seq	Kdm3 GLKD	ovary	αH3K9me2	25939857	24978229
ADQN-85	ChIP seq	Kdm3 GLKD	ovary	αH3K9me2	18977039	18211497
ADQN-86	ChIP seq	Kdm3 control sisters	ovary	αH3K9me3	17114226	15827951
ADQN-87	ChIP seq	Kdm3 control sisters	ovary	αH3K9me3	19588057	18078106
ADQN-88	ChIP seq	Kdm3 control sisters	ovary	αH3K9me3	23214725	21711093

ADQN-89	ChIP seq	Kdm3 GLKD	ovary	αH3K9me3	22208263	20666374
ADQN-90	ChIP seq	Kdm3 GLKD	ovary	αH3K9me3	24629347	23093308
ADQN-91	ChIP seq	Kdm3 GLKD	ovary	αH3K9me3	23634231	22259230
S1	ChIP seq	Kdm3 control sisters	ovary	no (INPUT for Rhino)	21871351	21491195
S2	ChIP seq	Kdm3 control sisters	ovary	αRhino	21389050	20979220
S3	ChIP seq	Kdm3 control sisters	ovary	αRhino	20097195	19719881
S4	ChIP seq	Kdm3 GLKD	ovary	no (INPUT for Rhino)	22166658	21797391
S5	ChIP seq	Kdm3 GLKD	ovary	αRhino	19800458	19457139
S6	ChIP seq	Kdm3 GLKD	ovary	αRhino	18726314	18378218

Table S3. Stability of *hfp* GLKD induced *BX2* conversion through subsequent generations. A complete and stable conversion was observed for seven lines established from seven single G2 *BX2* females and tested during 20 generations by cross with *P(lacZ)* males and ß-Galactosidase assay of the progeny ovaries. n = number of tested females.

Generation	Repression (%)	n
3	100	46
4	100	50
8	100	44
20	100	72

Table S4. Determination of regions presenting a differential expression of unique 23-29 nt RNAs. Coordinates, size, rank, gene content, ID, mean 23-29 nt expression, sigma, basemean and fold change are given. nc : not calculable.

Region	Size	Rank	Gene Content	ID	Mean Control (rpm)	Mean Mutant (rpm)	Sigma Control	Sigma Mutant	Base Mean	FC	log2 FC	log2 control	log2 mutant
chr2L:21766000-21948000	182000	R1	tsh	R1_tsh	17.4	531.2	2.9	22.4	274.3	30.6	4.9	4.1	9.1
chrX:4372000-4532000	160000	R2	bi	R2_bi	7.1	672.8	3.5	46.2	339.9	95.3	6.6	2.8	9.4
chrX:16101000-16223000	122000	R3	disco-r/disco	R3_disco-r/disco	7.6	272.5	1.7	25.8	140.0	35.9	5.2	2.9	8.1
chr2R:6503000-6613000	110000	R4	jing	R4_jing	7.7	566.5	2.8	24.7	287.1	73.4	6.2	2.9	9.1
chrX:7160000-7236000	76000	R5	CR44357	R5_CR44357	0.6	61.9	0.3	0.2	31.3	100.0	6.6	-0.7	6.0
chrX:8742000-8812000	70000	R6	Lim1	R6_Lim1	2.2	87.6	0.3	9.3	44.9	39.4	5.3	1.2	6.5
chr3R:4822000-4888000	66000	R7	opa/laf	R7_opa/laf	3.6	420.6	2.1	15.8	212.1	117.2	6.9	1.8	8.7
chr3L:22882000-22945000	63000	R8	BoYb	R8_BoYb	7.5	186.0	1.0	8.6	96.7	24.9	4.6	2.9	7.5
chrX:17767000-17820000	53000	R9	unc4/OdsH	R9_unc4/OdsH	2.3	75.8	0.8	8.8	39.1	32.3	5.0	1.2	6.2
chr4:491000-538000	47000	R10	zhf2	R10_zhf2	1.8	163.5	0.4	3.1	82.7	89.3	6.5	0.9	7.4
chr3L:23443000-23487000	44000	R-1	intergenic	R-1_intergenic	211.2	15.8	7.1	1.6	113.5	0.1	-3.7	7.7	4.0
chr2R:5718000-5753000	35000	R11	ap	R11_ap	2.5	90.0	1.0	4.7	46.2	36.7	5.2	1.3	6.5
chr3R:4480000-4515000	35000	R12	Fip1	R12_Fip1	2.1	67.7	0.7	2.2	34.9	32.0	5.0	1.1	6.1
chrX:518000-552000	34000	R13	Appl	R13_Appl	1.2	40.0	0.1	9.3	20.6	32.1	5.0	0.3	5.3
chr3L:22828000-22862000	34000	R14	jim	R14_jim	3.8	69.4	0.5	5.8	36.6	18.5	4.2	1.9	6.1
chr2L:21991000-22019000	28000	R15	CG31693	R15_CG31693	0.6	27.0	0.2	1.3	13.8	45.3	5.5	-0.7	4.8
chr2R:8636000-8664000	28000	R16	ptc	R16_ptc	1.3	24.8	0.1	6.6	13.1	19.4	4.3	0.4	4.6
chrX:2186000-2214000	28000	R17	CG14053	R17_CG14053	4.2	49.7	0.3	5.1	27.0	11.7	3.5	2.1	5.6
chrX:16231000-16258000	27000	R18	snRNA U5	R18_snRNA U5	1.0	25.1	0.2	1.0	13.0	25.6	4.7	0.0	4.6
chr3R:4341000-4366000	25000	R19	CG1090	R19_CG1090	0.4	27.8	0.1	2.5	14.1	64.2	6.0	-1.2	4.8
chr2L:22029000-22054000	25000	R20	CG31601	R20_CG31601	0.4	16.2	0.1	0.8	8.3	43.5	5.4	-1.4	4.0
chr3R:4914000-4938000	24000	R21	Cdep	R21_Cdep	1.6	18.3	0.0	1.4	10.0	11.3	3.5	0.7	4.2
chrX:9283000-9306000	23000	R22	lz	R22_lz	0.8	47.4	0.1	3.7	24.1	56.2	5.8	-0.2	5.6
chr2R:13019000-13042000	23000	R23	intergenic	R23_intergenic	1.7	22.0	0.2	2.3	11.8	13.0	3.7	0.8	4.5
chrX:8627000-8649000	22000	R24	oc	R24_oc	0.2	13.3	0.1	3.4	6.7	87.7	6.5	-2.7	3.7
chr3R:4278000-4300000	22000	R25	cpx	R25_cpx	2.6	28.4	0.3	3.3	15.5	10.7	3.4	1.4	4.8
chr3L:22750000-22772000	22000	R26	SpoCk	R26_SpoCk	2.9	28.4	0.8	1.5	15.7	9.7	3.3	1.5	4.8
chr3R:30249000-30268000	19000	R27	CG18404	R27_CG18404	2.6	27.1	0.6	3.1	14.9	10.3	3.4	1.4	4.8
chr3R:12357000-12376000	19000	R28	GstD9	R28_GstD9	16.9	131.0	0.9	18.1	74.0	7.7	3.0	4.1	7.0
chrX:4599000-4617000	18000	R29	CR32773	R29_CR32773	0.2	6.2	0.1	1.2	3.2	37.4	5.2	-2.6	2.6
chr3L:371000-389000	18000	R30	trh	R30_trh	0.3	11.4	0.2	2.9	5.8	37.2	5.2	-1.7	3.5
chrX:22832000-22850000	18000	R31	fog	R31_fog	0.8	12.6	0.2	1.8	6.7	15.8	4.0	-0.3	3.7
chr3L:1448000-1466000	18000	R32	rho	R32_rho	0.5	7.8	0.2	0.7	4.1	15.0	3.9	-0.9	3.0
chr3L:21768000-21786000	18000	R33	Syn1	R33_Syn1	0.9	12.3	0.1	1.4	6.6	14.0	3.8	-0.2	3.6
chr2L:19013000-19030000	17000	R34	CR43700	R34_CR43700	0.2	9.2	0.0	0.3	4.7	44.1	5.5	-2.3	3.2
chrX:6009000-6026000	17000	R35	mab21	R35_mab21	0.2	8.7	0.2	0.8	4.5	39.7	5.3	-2.2	3.1
chr3R:4241000-4258000	17000	R36	TwdlG	R36_TwdlG	1.6	50.3	0.3	0.9	25.9	31.7	5.0	0.7	5.7

chr3L:1375000-1392000	17000	R37	Ptp61F	R37_Ptp61F	0.9	13.6	0.5	0.8	7.2	15.6	4.0	-0.2	3.8
chr3L:19481000-19498000	17000	R38	CG9449	R38_CG9449	1.2	15.2	0.2	2.7	8.2	12.6	3.7	0.3	3.9
chr2L:20231000-20247000	16000	R39	intergenic	R39_intergenic	0.3	13.3	0.1	1.8	6.8	47.3	5.6	-1.8	3.7
chrX:568000-584000	16000	R40	Appl	R40_Appl	1.6	16.4	0.3	2.4	9.0	10.0	3.3	0.7	4.0
chr3L:64000-79000	15000	R-2	Lsp1gamma	R-2_Lsp1gamma	34.8	1.0	7.7	0.2	17.9	0.0	-5.2	5.1	-0.1
chr3R:6662000-6677000	15000	R41	lab	R41_lab	0.3	58.9	0.3	12.4	29.6	181.3	7.5	-1.6	5.9
chr2L:20257000-20272000	15000	R42	CG17570	R42_CG17570	0.1	4.9	0.1	0.6	2.5	53.3	5.7	-3.5	2.3
chrX:766000-781000	15000	R43	fz3	R43_fz3	0.6	22.3	0.1	0.3	11.5	36.0	5.2	-0.7	4.5
chr3R:3897000-3911000	14000	R-3	intergenic	R-3_intergenic	592.2	46.0	20.5	6.1	319.1	0.1	-3.7	9.2	5.5
chr3L:22078000-22092000	14000	R44	msopa	R44_msopa	0.1	8.1	0.0	0.6	4.1	56.3	5.8	-2.8	3.0
chr2L:19071000-19085000	14000	R45	Lim3	R45_Lim3	0.2	7.2	0.1	0.7	3.7	41.4	5.4	-2.5	2.8
chr3L:21202000-21216000	14000	R46	CG10508	R46_CG10508	0.6	15.8	0.2	0.9	8.2	25.7	4.7	-0.7	4.0
chr4:402000-416000	14000	R47	intergenic	R47_intergenic	0.6	14.0	0.1	1.9	7.3	24.9	4.6	-0.8	3.8
chr3R:18911000-18925000	14000	R48	Xrp1	R48_Xrp1	3.6	33.1	0.7	3.1	18.3	9.2	3.2	1.8	5.0
chr3R:3376000-3389000	13000	R-4	intergenic	R-4_intergenic	232.8	34.2	6.5	2.6	133.5	0.1	-2.8	7.9	5.1
chrX:8608000-8621000	13000	R49	intergenic	R49_intergenic	0.1	11.9	0.1	3.6	6.0	96.0	6.6	-3.0	3.6
chr3R:4894000-4907000	13000	R50	CR45580	R50_CR45580	0.7	58.6	0.4	2.6	29.6	81.7	6.4	-0.5	5.9
chr2R:8784000-8797000	13000	R51	intergenic	R51_intergenic	0.3	7.1	0.2	2.1	3.7	25.6	4.7	-1.9	2.8
chr3R:4449000-4462000	13000	R52	CG31522	R52_CG31522	0.4	8.0	0.1	1.2	4.2	21.7	4.4	-1.4	3.0
chrX:21504000-21517000	13000	R53	CR45082	R53_CR45082	0.5	7.0	0.2	1.0	3.7	15.2	3.9	-1.1	2.8
chr2L:22059000-22071000	12000	R54	CG42597	R54_CG42597	0.1	4.4	0.0	0.5	2.2	33.3	5.1	-2.9	2.1
chr3L:16762000-16774000	12000	R55	Nrt	R55_Nrt	0.2	4.8	0.1	0.9	2.5	30.7	4.9	-2.7	2.3
chrX:22719000-22731000	12000	R56	CR44997	R56_CR44997	1.2	12.2	0.3	0.4	6.7	10.0	3.3	0.3	3.6
chr3R:3989000-4000000	11000	R-5	intergenic	R-5_intergenic	87.9	5.2	4.9	0.3	46.6	0.1	-4.1	6.5	2.4
chr3R:6964000-6975000	11000	R57	Antp	R57_Antp	0.2	15.6	0.2	0.5	7.9	98.7	6.6	-2.7	4.0
chr2L:21200000-21211000	11000	R58	clumsy	R58_clumsy	0.1	6.7	0.0	1.1	3.4	45.3	5.5	-2.8	2.7
chrX:20629000-20640000	11000	R59	intergenic	R59_intergenic	0.2	6.9	0.0	0.2	3.5	41.4	5.4	-2.6	2.8
chr3L:24555000-24566000	11000	R-6	intergenic	R-6_intergenic	507.4	16.5	20.1	0.8	262.0	0.0	-4.9	9.0	4.0
chr2L:21971000-21982000	11000	R60	CG2528	R60_CG2528	0.3	7.5	0.2	0.6	3.9	29.5	4.9	-2.0	2.9
chr3L:21251000-21262000	11000	R61	Eip78C	R61_Eip78C	1.3	26.7	0.3	3.4	14.0	21.0	4.4	0.3	4.7
chrX:18617000-18628000	11000	R62	wgn	R62_wgn	0.3	5.5	0.1	0.3	2.9	16.8	4.1	-1.6	2.4
chr2L:14477000-14488000	11000	R63	CR44731	R63_CR44731	0.8	7.6	0.3	1.1	4.2	9.2	3.2	-0.3	2.9
chr2L:492000-503000	11000	R64	ush	R64_ush	0.7	5.4	0.1	0.9	3.0	7.8	3.0	-0.5	2.4
chrX:8728000-8738000	10000	R65	intergenic	R65_intergenic	0.1	6.6	0.0	1.0	3.3	122.9	6.9	-4.2	2.7
chr3L:22274000-22284000	10000	R66	Sprk79D	R66_Sprk79D	0.3	17.3	0.1	1.9	8.8	51.7	5.7	-1.6	4.1
chrX:7141000-7151000	10000	R67	CR32730	R67_CR32730	0.1	5.2	0.1	0.2	2.7	37.6	5.2	-2.8	2.4
chr2R:11391000-11401000	10000	R68	sprt	R68_sprt	0.1	3.9	0.1	0.8	2.0	34.9	5.1	-3.2	1.9
chrX:22513000-22523000	10000	R69	CG17600	R69_CG17600	0.3	7.0	0.1	0.6	3.7	22.1	4.5	-1.7	2.8
chr3R:3878000-3887000	9000	R-7	intergenic	R-7_intergenic	141.2	15.2	6.9	1.0	78.2	0.1	-3.2	7.1	3.9
chrX:5601000-5610000	9000	R70	Vsx1	R70_Vsx1	0.0	3.3	0.0	1.1	1.6	nc	nc	nc	1.7
chr3L:278000-287000	9000	R71	RhoGEF3	R71_RhoGEF3	0.0	3.1	0.0	0.2	1.6	78.2	6.3	-4.7	1.6
chr3L:9012000-9021000	9000	R72	Doc2	R72_Doc2	0.0	3.3	0.1	0.7	1.7	77.2	6.3	-4.5	1.7
chrX:22786000-22795000	9000	R73	CR44997	R73_CR44997	0.2	8.6	0.2	0.6	4.4	51.6	5.7	-2.6	3.1

chrX:22693000-22702000	9000	R74	CR44997	R74_CR44997	0.2	4.1	0.1	0.9	2.1	19.6	4.3	-2.3	2.0
chr2L:20128000-20137000	9000	R75	CG10651	R75_CG10651	0.3	4.3	0.1	0.2	2.3	16.1	4.0	-1.9	2.1
chr2L:16477000-16485000	8000	R76	dac	R76_dac	0.0	3.4	0.0	0.8	1.7	85.0	6.4	-4.6	1.8
chrX:17755000-17763000	8000	R77	intergenic	R77_intergenic	0.1	4.8	0.0	0.7	2.4	77.1	6.3	-4.0	2.3
chrX:21482000-21490000	8000	R78	CR45511	R78_CR45511	0.1	3.6	0.0	0.8	1.8	71.0	6.2	-4.3	1.8
chr2R:25084000-25092000	8000	R79	lov	R79_lov	0.0	2.9	0.1	0.3	1.5	69.7	6.1	-4.6	1.5
chr2R:4874000-4882000	8000	R-8	intergenic	R-8_intergenic	20.2	2.8	2.8	0.8	11.5	0.1	-2.8	4.3	1.5
chr2L:21185000-21193000	8000	R80	Ret	R80_Ret	0.1	3.5	0.1	0.7	1.8	59.0	5.9	-4.1	1.8
chr3R:31158000-31166000	8000	R81	stops	R81_stops	0.0	2.0	0.0	0.2	1.0	54.3	5.8	-4.8	1.0
chr3L:21357000-21365000	8000	R82	CG32440	R82_CG32440	0.3	11.3	0.1	0.9	5.8	42.2	5.4	-1.9	3.5
chrX:356000-364000	8000	R83	y	R83_y	0.1	2.7	0.0	0.3	1.4	30.9	4.9	-3.5	1.4
chrX:1381000-1389000	8000	R84	CG32813	R84_CG32813	0.3	7.5	0.1	1.1	3.9	22.7	4.5	-1.6	2.9
chr2R:6058000-6066000	8000	R85	CCHa2	R85_CCHa2	0.1	2.9	0.1	0.1	1.5	19.5	4.3	-2.8	1.5
chr2R:24950000-24958000	8000	R86	CG12851	R86_CG12851	0.1	2.3	0.1	0.4	1.2	18.5	4.2	-3.0	1.2
chr2R:8327000-8335000	8000	R87	pdm3	R87_pdm3	0.2	2.8	0.0	0.2	1.5	16.4	4.0	-2.6	1.5
chr2R:5790000-5798000	8000	R88	Or42a	R88_Or42a	0.3	4.2	0.2	0.3	2.2	14.6	3.9	-1.8	2.1
chr2L:21709000-21717000	8000	R89	nolo	R89_nolo	0.4	5.2	0.1	0.7	2.8	14.5	3.9	-1.5	2.4
chrX:868000-876000	8000	R90	CG11664	R90_CG11664	0.3	4.2	0.1	1.7	2.3	13.9	3.8	-1.7	2.1
chr4:385000-393000	8000	R91	dati	R91_dati	0.4	4.5	0.3	0.4	2.4	11.4	3.5	-1.3	2.2
chr2R:23622000-23630000	8000	R92	intergenic	R92_intergenic	0.2	2.2	0.1	0.6	1.2	11.0	3.5	-2.3	1.1
chrX:2790000-2797000	7000	R100	w(BX2)	R100_w(BX2)	6.4	283.0	0.9	44.7	144.7	44.0	5.5	2.7	8.1
chrX:10619000-10626000	7000	R101	Rhab9Db	R101_Rhab9Db	0.1	4.9	0.0	0.9	2.5	42.5	5.4	-3.1	2.3
chr3R:4778000-4785000	7000	R102	CG17387	R102_CG17387	0.1	3.7	0.0	0.5	1.9	41.9	5.4	-3.5	1.9
chr2L:22075000-22082000	7000	R103	ttm3	R103_ttm3	0.1	3.3	0.0	0.3	1.7	36.4	5.2	-3.5	1.7
chr3L:21237000-21244000	7000	R104	Eip78C	R104_Eip78C	0.3	9.7	0.2	1.6	5.0	28.2	4.8	-1.5	3.3
chr3R:16217000-16224000	7000	R105	CR45643	R105_CR45643	0.1	2.3	0.1	0.3	1.2	27.0	4.8	-3.6	1.2
chr3R:4520000-4527000	7000	R106	CG34357	R106_CG34357	0.3	5.5	0.1	0.8	2.9	21.5	4.4	-2.0	2.5
chr2R:11486000-11493000	7000	R107	inv	R107_inv	0.2	2.8	0.1	0.5	1.5	17.6	4.1	-2.6	1.5
chrX:1534000-1541000	7000	R108	Mur2B	R108_Mur2B	0.3	5.1	0.1	1.4	2.7	17.2	4.1	-1.8	2.3
chr2L:435000-442000	7000	R109	ex	R109_ex	0.2	2.9	0.0	0.5	1.5	13.9	3.8	-2.3	1.5
chr2L:9774000-9781000	7000	R110	ppk	R110_ppk	0.4	4.6	0.2	0.8	2.5	13.2	3.7	-1.5	2.2
chr2R:7246000-7253000	7000	R111	Gadd45a	R111_Gadd45a	4.8	51.4	0.6	7.2	28.1	10.7	3.4	2.3	5.7
chr2R:24891000-24898000	7000	R112	Ance-5	R112_Ance-5	0.3	3.7	0.1	1.0	2.0	10.7	3.4	-1.5	1.9
chr2L:21607000-21614000	7000	R113	intergenic	R113_intergenic	0.9	8.1	0.0	0.4	4.5	9.3	3.2	-0.2	3.0
chr3L:14614000-14621000	7000	R114	shd	R114_shd	0.5	3.5	0.1	1.1	2.0	7.5	2.9	-1.1	1.8
chr3L:21269000-21276000	7000	R115	AcCoAS	R115_AcCoAS	3.3	6.5	0.5	0.4	4.9	2.0	1.0	1.7	2.7
chr3R:16138000-16145000	7000	R-9	Sb	R-9_Sb	10.1	0.1	2.4	0.1	5.1	0.0	-6.7	3.3	-3.3
chr2L:19515000-19522000	7000	R93	CG10132	R93_CG10132	0.0	2.9	0.0	0.8	1.4	250.1	8.0	-6.5	1.5
chr3R:31263000-31270000	7000	R94	ppk24	R94_ppk24	0.0	3.9	0.0	0.3	2.0	172.5	7.4	-5.5	2.0
chrX:7242000-7249000	7000	R95	CR44357	R95_CR44357	0.0	2.8	0.0	0.3	1.4	121.6	6.9	-5.5	1.5
chrX:5533000-5540000	7000	R96	Vsx2	R96_Vsx2	0.0	2.7	0.0	0.5	1.4	69.4	6.1	-4.7	1.5
chr2R:14781000-14788000	7000	R97	kn	R97_kn	0.1	3.3	0.1	0.3	1.7	48.9	5.6	-3.9	1.7
chr3R:5437000-5444000	7000	R98	CG14669	R98_CG14669	0.1	3.7	0.0	1.0	1.9	48.1	5.6	-3.7	1.9

chrX:9310000-9317000	7000	R99	CR44534	R99_CR44534	0.1	4.6	0.1	0.2	2.4	46.8	5.5	-3.3	2.2
chr3R:3972000-3978000	6000	R-10	intergenic	R-10_intergenic	10.2	1.4	0.5	0.1	5.8	0.1	-2.9	3.4	0.4
chr3R:10638000-10644000	6000	R-11	CR44018	R-11_CR44018	5.0	0.0	0.5	0.0	2.5	0.0	nc	2.3	nc
chr2R:7229000-7235000	6000	R116	Or43a	R116_Or43a	0.0	2.0	0.0	0.5	1.0	170.8	7.4	-6.5	1.0
chr3L:18505000-18511000	6000	R117	intergenic	R117_intergenic	0.0	2.9	0.0	0.2	1.4	125.4	7.0	-5.5	1.5
chr3R:7815000-7821000	6000	R118	CG34384	R118_CG34384	0.1	6.0	0.1	0.9	3.0	104.9	6.7	-4.1	2.6
chr2L:20833000-20839000	6000	R119	Spn38F	R119_Spn38F	0.2	9.1	0.0	0.8	4.7	46.6	5.5	-2.4	3.2
chr2R:21175000-21181000	6000	R120	Pu	R120_Pu	0.3	8.5	0.2	1.7	4.4	25.4	4.7	-1.6	3.1
chr3L:14272000-14278000	6000	R121	fz	R121_fz	0.4	9.9	0.2	1.0	5.2	22.8	4.5	-1.2	3.3
chr3R:4531000-4537000	6000	R122	CG34357	R122_CG34357	0.8	9.4	0.2	1.9	5.1	11.5	3.5	-0.3	3.2
chrX:22545000-22551000	6000	R123	CG17601	R123_CG17601	0.9	7.0	0.0	0.5	4.0	8.1	3.0	-0.2	2.8

Table S5. intron/exon metric.

	Kdm3 GLKD #1	Kdm3 GLKD #2	Kdm3 GLKD #3	Mean	SD	Total sequence size (kb)	RPKM
Library	ALBA-28	ALBA-29	ALBA-30				
Normalization factor	0.05	0.03	0.04				
Unique mappers 23-29 nt /whole genome (dm6)	1287136	1996135	1637207				
Unique mappers 23-29 nt /whole genome (dm6 rpm)	61965.86	67477.14	64040.54	64494.51	2783.55	143700.00	**0.45**
Unique mappers 23-29 nt /123 regions	99199	146587	130517				
Unique mappers 23-29 nt /123 regions (rpm)	4775.68	4955.21	5105.27	4945.39	165.01	2382.00	**2.08**
Unique mappers 23-29 nt /123 regions /introns	56716	83510	74282				
Unique mappers 23-29 nt /123 regions /introns (rpm)	2730.45	2822.96	2905.59	2819.67	87.62	1181.94	**2.39**
Unique mappers 23-29 nt /123 regions /exons	6121	8911	8239				
Unique mappers 23-29 nt /123 regions /exons (rpm)	294.68	301.23	322.27	306.06	14.42	154.90	**1.98**
Unique mappers 23-29 nt /13 regions	72311	104273	92341				
Unique mappers 23-29 nt /13 regions (rpm)	3481.23	3524.83	3611.99	3539.35	66.58	949.00	**3.73**
Unique mappers 23-29 nt /13 regions /introns	45443	65464	57915				
Unique mappers 23-29 nt /13 regions /introns (rpm)	2187.74	2212.94	2265.39	2222.02	39.61	498.28	**4.46**
Unique mappers 23-29 nt /13 regions /exons	3794	5367	4777				
Unique mappers 23-29 nt /13 regions /exons (rpm)	182.65	181.43	186.86	183.64	2.85	52.63	**3.49**

Table S6. Statistical analyses of RT-qPCR experiments. The number of biological replicates is given and then the appropriate method used as the following Post-hoc test.

	Number of biological replicates			
	Kdm3+/+ Moon+/+	*Kdm3GLKD Moon+/+*	*Kdm3+/+ Moon-/-*	*Kdm3GLKD Moon-/-*
eEF5	6	4	3	4
bifid	6	5	3	4
disco	6	5	3	4
disco-R	6	3	3	4
lab	6	4	3	4
lim1	6	5	3	3
oc	6	5	3	4
opa	6	4	3	3
tsh	6	4	3	4
unc-4	6	3	3	4

	***p*-value**	**Method**	**Following Post-hoc test**
fig3e_eEF5	0.064	ANOVA	_
fig3e_bifid	0.00018	ANOVA	TukeyHSD
fig3e_disco	0.000284	Welch ANOVA	Games Howell
fig3e_discoR	1.05e-06	ANOVA	TukeyHSD
fig3e_lab	8.47e-05	Welch ANOVA	Games Howell
fig3e_Lim1	0.000279	Welch ANOVA	Games Howell
fig3e_oc	0.002	Welch ANOVA	Games Howell
fig3e_opa	1.54e-12	ANOVA	TukeyHSD
fig3e_tsh	0.00042	Welch ANOVA	Games Howell
fig3e_unc4	2.74e-09	ANOVA	TukeyHSD

TukeyHSD tests					
**group1**	**group2**	***p*.adj**			
		**bifid**	**discoR**	**opa**	**unc4**
Kdm3+/+_Moon+	Kdm3GLKD_Moon+/+	0.000255	5.04e-07	1.43e-12	3.56e-09
Kdm3+/+_Moon+/+	Kdm3+/+_Moon-/-	0.998	0.0704	0.997	0.999
Kdm3+/+_Moon+/+	Kdm3GLKD_Moon-/-	0.981	0.00242	3.19e-06	0.978
Kdm3GLKD_Moon+/+	Kdm3+/+_Moon-/-	0.00206	4.41e-05	5.58e-12	1.85e-08
Kdm3GLKD_Moon+/+	Kdm3GLKD_Moon-/-	0.00132	0.000158	9.1e-10	1.03e-08
Kdm3+/+_Moon-/-	Kdm3GLKD_Moon-/-	0.999	0.509	1.79e-05	0.995

Games Howell tests						
**group1**	**group2**	***p*.adj**				
		**disco**	**lab**	**Lim1**	**oc**	**tsh**
Kdm3+/+_Moon-/-	Kdm3+/+_Moon+/+	0.229	0.929	0.925	0.746	0.035
Kdm3+/+_Moon-/-	Kdm3GLKD_Moon-/-	0.393	0.048	0.954	0.997	0.017
Kdm3+/+_Moon-/-	Kdm3GLKD_Moon+/+	0.00027	0.023	0.000115	0.003	0.165
Kdm3+/+_Moon+/+	Kdm3GLKD_Moon-/-	0.031	2.08e-06	0.997	0.297	0.004
Kdm3+/+_Moon+/+	Kdm3GLKD_Moon+/+	0.000455	0.023	0.000279	0.004	0.1
Kdm3GLKD_Moon-/-	Kdm3GLKD_Moon+/+	0.000367	0.031	0.00025	0.004	0.32

Table S7. Genome wide ChIP-seq analyses using H3K9me2, H3K9me3 and Rhino antibodies. MACS2 analyses gave the number of significant enriched peaks for both H3K9 marks and Rhino compared to their respective input. The total length of enriched sequences and their proportion in relation to the total size of the *Drosophila melanogaster* genome (%) are given. BED intersect analyses allowed to determine overlap coordinates and to define co-enriched regions.

	**MACS2 peaks**		**Size (pb)**		**% D.mel genome**		**FC**
**Enrichment (IP/INPUT)**	Control	*Kdm3* GLKD	Control	*Kdm3* GLKD	Control	*Kdm3* GLKD	
H3K9me2	3983	9564	28029688	44185495	20.94	33.00	1.5764
H3K9me3	2946	5282	23497658	27812726	17.55	20.77	1.1836
Rhino	467	1882	244106	1222674	0.18	0.91	5.0088
Overlap			Size (pb)		% D.mel genome		
H3K9me2	H3K9me3	Rhino	Control	*Kdm3* GLKD	Control	*Kdm3* GLKD	
-	-	-	105262523	84963645	78.62	63.46	0.8072
+	+	-	22694917	22676421	16.95	16.94	0.9992
+	-	-	5104765	20670476	3.81	15.44	4.0493
-	+	-	574297	4347392	0.43	3.03	7.5699
+	+	+	228444	788729	0.17	0.59	3.4526
-	-	+	14100	383892	0.01	0.29	27.2264
+	-	+	1562	49869	0.00	0.04	31.9264
-	+	+	0	184	0.00	0.00	na

Table S8. Chromatin enrichment of the 123 additional piRNA clusters in control and *Kdm3* GLKD ovaries. MACS2 analyses revealed the presence of H3K9me2, H3K9me3 and/or Rhino enrichments upon the 123 additional piRNA producing regions in *Kdm3* GLKD and in these regions in the control condition. The cumulative size of these regions is given.

	**Size (pb)**		**% additional piRNA clusters sequences**		**Fold change**		
	Control	Kdm3 GLKD	Control	Kdm3 GLKD			
H3K9me2	203141	1771831	8.53	74.38	8.7222		
H3K9me3	95781	747655	4.02	31.39	7.8059		
Rhino	0	508852	0.00	21.36	na		
**Overlap**			**Size (pb)**		**% additional piRNA clusters sequences**		**Fold change**
H3K9me2	H3K9me3	Rhino	Control	Kdm3 GLKD	Control	Kdm3 GLKD	
-	-	-	2167497	373694	90.99	15.69	0.1724
+	+	-	84419	448252	3.54	18.82	5.3098
+	-	-	118722	1020055	4.98	42.82	8.5920
-	+	-	11362	31147	0.48	1.31	2.7413
+	+	+	0	268072	0.00	11.25	na
-	-	+	0	205144	0.00	8.61	na
+	-	+	0	35452	0.00	1.49	na
-	+	+	0	184	0.00	0.01	na

Table S9. piRNA production linked to the chromatin state of the 123 additional piRNA clusters. The 23-29 nt (unique mappers) production was obtained using ovarian small RNA-seq analyses and the rpm value was calculated using normalization factors estimated from each library depth.

	**Size (kbp)**		**Total size %**		**Mean**		**Std**		**Density (RPKM)**		**Density std**	
	**Control**	***Kdm3* GLKD**	**Control**	***Kdm3* GLKD**	**Control**	***Kdm3* GLKD**	**Control**	***Kdm3* GLKD**	**Control**	***Kdm3* GLKD**	**Control**	***Kdm3* GLKD**
**H3K9me2+H3K9me3+Rhi**	0.00	268.07	0.00	11.25	0.00	1430.37	0.00	39.01	0.00	5.34	na	0.15
**Rhi (only)**	0.00	205.14	0.00	8.61	0.00	723.05	0.00	34.75	0.00	3.52	na	0.17
**H3K9me2+Rhi**	0.00	35.45	0.00	1.49	0.00	124.94	0.00	3.95	0.00	3.52	na	0.11
**H3K9me3 (only)**	11.36	31.15	0.48	1.31	0.94	83.10	0.11	8.19	0.08	2.67	0.01	0.26
**H3K9me2 (only)**	118.72	1020.06	4.98	42.82	15.92	1786.01	1.09	68.91	0.13	1.75	0.01	0.07
**H3K9me2+H3K9me3**	84.42	448.25	3.54	18.82	18.92	758.60	0.85	20.41	0.22	1.69	0.01	0.05
**H3K9me3+Rhi**	0.00	0.18	0.00	0.01	0.00	0.12	0.00	0.04	0.00	0.65	na	0.19
**none**	2167.50	373.69	90.99	15.69	118.07	37.11	15.57	12.81	0.05	0.10	0.01	0.03
**Total**	2382.00	2382.00			153.84	4943.29	17.33	157.29	0.06	2.08	0.01	0.07

Table S10. DESeq2 analysis on RNA-seq data from embryos issued from *Kdm3* GLKD or *w* GLKD females as control. Genes presented here correspond to those that were tested by RT-qPCR experiments (Fig. 5h).

FBgn	basemean	log2FC	St.Err	Wald	pval	padj	coordinates	gene name
FBgn0002522	231.80395	-1.49766	0.53284	-2.81072	0.00494	0.02157	chr3R:6661427-6678590	*lab*
FBgn0003002	3552.53658	-1.33317	0.47574	-2.80230	0.00507	0.02198	chr3R:4852813-4869979	*opa*
FBgn0003866	4561.83912	-0.93902	0.38352	-2.44841	0.01435	0.04732	chr2L:21828593-21837011	*tsh*
FBgn0004102	1587.16712	-1.06745	0.43693	-2.44306	0.01456	0.04786	chrX:8630159-8650681	*oc*
FBgn0000459	736.61079	-1.06092	0.44011	-2.41057	0.01593	0.05104	chrX:16210598-16216750	*disco*
FBgn0026411	209.46303	-1.04308	0.54162	-1.92587	0.05412	0.12486	chrX:8756539-8805804	*Lim1*
FBgn0285879	374.92266	-0.85580	0.53724	-1.59294	0.11117	0.21168	chrX:16118067-16148977	*disco-r*
FBgn0000179	473.87677	-0.67220	0.51513	-1.30491	0.19192	0.31298	chrX:4412856-4485647	*bi*
FBgn0024184	89.39502	-0.80753	0.64454	-1.25288	0.21025	0.33246	chrX:17768569-17778708	*unc-4*

Table S11. List of primers used.

purpose	name	sequence 5’→3’
hfp shRNA efficiency	hfp3UTRfor	GCCAGTCCGAAAGCCAAC
“	hfp3UTRrev	GACAATTGCTTCCCTCGCTG

Kdm3 shRNA efficiency	Kdm3_CDSfor	AGAGATTTGCGACGTGTGTG
“	Kdm3_CDSrev	AATCCCTCCTTGCGATCTTT
EGFP cloning	BE5EGFP	AAGGATCCGAATTCATGGTGAGCAAGGGCGAGGAG
“	B3EGFP	AAGGATCCTTACTTGTACAGCTGTCCATG
Kdm3 CDS cloning	E5JHD	AAGAATTCATGTCGCAAAAAGAATTGGCG
“	E3JHD	AAGAATTCATCTGCATTTAGCTTGGTTAATTCC
Kdm3 RT-qPCR	Rpl32for	CCGCTTCAAGGGACAGTATCTG
“	Rpl32rev	ATCTCGCCGCAGTAAACGC
“	Kdm3-RA_Splicedfor	TCAAGCCATTACACAACATGCA
“	Kdm3-RA_Splicedrev	TGAGGTCGAGGATATGGTGC
“	Kdm3-RB_Splicedfor	TGCACATGTTTAAATTTCCCGA
“	Kdm3-RB_Splicedrev	CAACCAGAGCCGCCAATTC
“	Kdm3-RA+RB_Unsplicedfor	CGACTGAAGTTTACACGGGC
“	Kdm3-RA+RB_Unsplicedrev	CAGTGCAGCGCGTTGTTTT
“	Kdm3-RB_Unsplicedfor	ACCACCTCAAACCACGATCC
“	Kdm3-RB_Unsplicedrev	ACCAGAGCCGCCAATTCTTT
RNA IP RTqPCR	eEF5for	TAACATGGATGTGCCCAATG
“	eEF5rev	AACGCAATTGTTCACCCAAT
“	Kdm3_CDSfor	AGAGATTTGCGACGTGTGTG
“	Kdm3_CDSrev	AATCCCTCCTTGCGATCTTT
“	Otu-1for	TGGGTGTAGAGAACGACGTG
“	Otu-1rev	ATACATGCCATTCGGTGGTT
“	tra2-1for	TCCTTGTGTCCGGATGAAGT
“	tra2-1rev	AAAAGCCAATGGATCGGGAG
“	lacZ1 (BX2)for	GAGAATCCGACGGGTTGTTA
“	lacZ1 (BX2)rev	AAATTCAGACGGCAAACGAC
RTqPCR	bifor	ACAAACAACAGTTACAGCAGC
“	birev	GGCAAAACGTCGAAAGTGT
“	discofor	TTCCACATCCTGTTCCTGCT
“	discorev	TTTCAGCCTTCACAAGCGT
“	disco-Rfor	CTTTAGAGCACCCTTGTCGC

“	disco-Rrev	ACCCAGTTCATCAATCCGGT
“	labfor	TGAGGTTCAGGTTAGGAGGC
“	labrev	GAGCGACTTGGGGTAGATGA
“	Lim1for	GCATTGAAGGGCATCGGTC
“	Lim1rev	GGACGCCAAGTTCGAGTTC
“	ocfor	ATCACCACCGGCATCCTTTA
“	ocrev	CCTCAATTTGGGCGTCGG
“	opafor	CATGAACCCGCTGAACCATT
“	oparev	TCTCCCACTCTCAATACGCC
“	tshfor	CAATGCCAACTCAGATGCCA
“	tshrev	GCCTTGTCGCACACTTTACA
“	unc-4for	CCCTCTCGCTCTTTCGCT
“	unc-4rev	TCGGTTTGCTTTCGTTTGGT
